# Not So Bioorthogonal Chemistry

**DOI:** 10.1021/jacs.4c15986

**Published:** 2025-02-28

**Authors:** Dominik Schauenburg, Tanja Weil

**Affiliations:** † Max Planck Institute for Polymer Research, Ackermannweg 10, 55128 Mainz, Germany; ‡ Department of Instructive Biomaterials Engineering, MERLN Institute for Technology-Inspired Regenerative Medicine, Maastricht University, 6229 ER Maastricht, The Netherlands

## Abstract

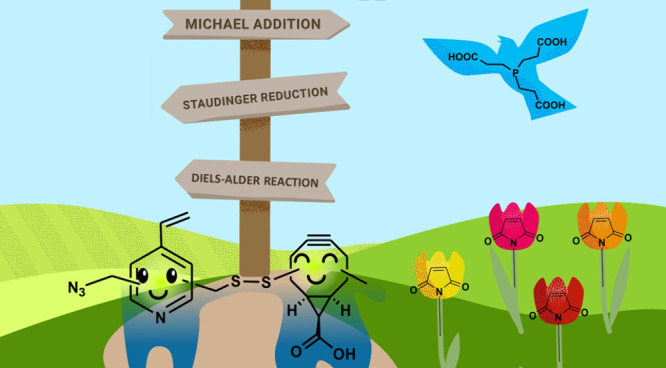

The advent of bioorthogonal chemistry has transformed scientific research, offering a powerful tool for selective and noninvasive labeling of (bio)molecules within complex biological environments. This innovative approach has facilitated the study of intricate cellular processes, protein dynamics, and interactions. Nevertheless, a number of challenges remain to be addressed, including the need for improved reaction kinetics, enhanced biocompatibility, and the development of a more diverse and orthogonal set of reactions. While scientists continue to search for veritable solutions, bioorthogonal chemistry remains a transformative tool with a vast potential for advancing our understanding of biology and medicine. This Perspective offers insights into reactions commonly classified as “bioorthogonal”, which, however, may not always demonstrate the desired selectivity regarding the interactions between their components and the additives or catalysts used under the reaction conditions.

## Bioorthogonal Chemistry: A Laudatory Speech

**Figure 1:**
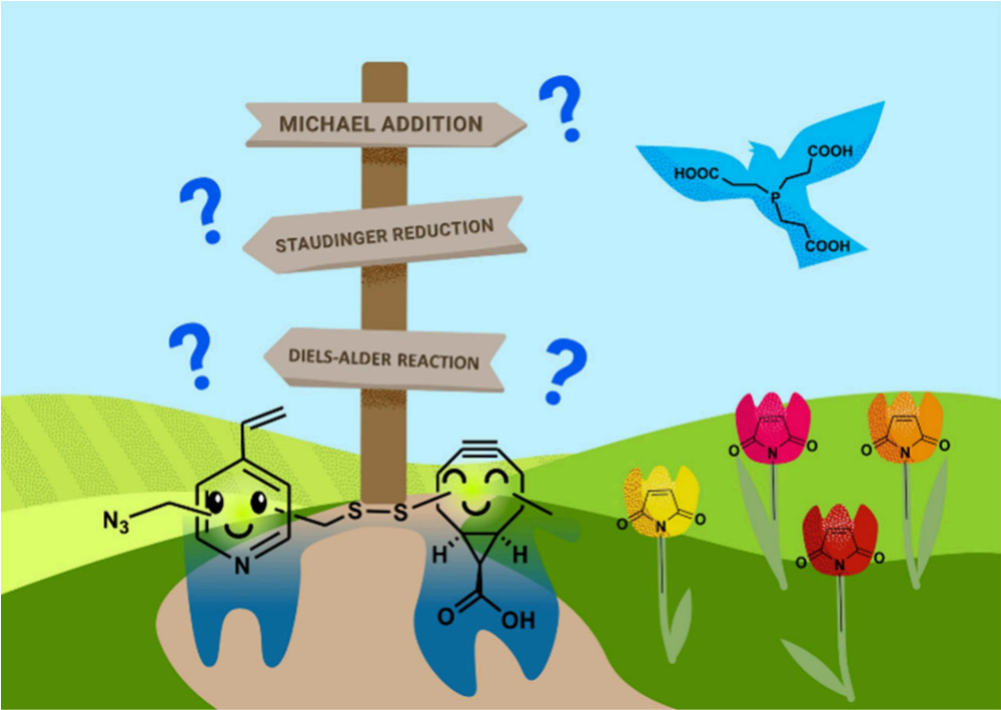
“Quo vadis?” Exogenous functional groups (bioconjugation or “click” handles), which can undergo (unintended) side reactions with endogenous or exogenous functionalities.

In the past few decades, bioorthogonal reactions, which proceed in complex biological environments without the formation of side products, have emerged as a powerful tool to solve diverse research challenges.^1,2^ Due to their relative simplicity and robustness, they are applied in chemical, biophysical, biochemical, and medicinal research.^3^ These reactions are distinguished by their capacity to occur in intricate biological environments without interfering with native processes; they are considered biocompatible and thus have been extensively utilized for biomolecule labeling (Figure 1).^4^ In the realm of proteins and DNA, bioorthogonal reactions enable site-selective bioconjugation, facilitating the investigation of protein dynamics, interactions, and cellular localization.^3,5^ Similarly, these reactions have proven invaluable for site-specific modification and labeling of nucleic acids, contributing to studies on gene expression and genome dynamics.^3,6−9^

Cell surface engineering has greatly benefited from bioorthogonal chemistry, enabling the precise modification of cell surface receptors as well as the metabolic labeling of polysaccharides, a process in which bioorthogonal chemical tags are incorporated into cellular biomolecules through the cell’s natural metabolic pathways, allowing for selective tracking and modification.^10−12^ This capability has enhanced our understanding of cell adhesion, migration, and communication.^13,14^ The well-known “click” reactions, introduced by K. Barry Sharpless and Morten Meldal, have revolutionized chemical biology and have found many applications in bioconjugation, drug discovery, and material science.^15,16^ Sharpless defined “click” reactions as a set of transformations that are modular, are wide in scope, give very high yields, and generate only inoffensive byproducts.^17^

Carolyn Bertozzi expanded this work, adapting “click” chemistry for bioorthogonal applications, enabling selective reactions in living systems without interfering with biomolecules or biological processes.^18−21^ To meet the essential criteria for successful bioorthogonality, several requirements must be fulfilled.^21−23^ First, biocompatibility is crucial, necessitating reaction conditions that are mild, occurring at physiological conditions and temperatures and under near-neutral pH conditions without organic solvents. The reagents involved should ideally be nontoxic to cells and organisms, and the reaction should be inert to reactive groups present in endogenous biomolecules such as thiols, amines, and alcohols, among others, minimizing background interference.^17^ Orthogonality is another key requirement, meaning that the bioorthogonal reaction operates exclusively on one functional group in the presence of other reactive groups, i.e., present on proteins or nucleic acids, thus ensuring high chemoselectivity in the labeling process.^24^ Fast kinetics are essential to ensure that the reaction proceeds efficiently at low concentrations within the dynamic timeframes of biological processes.^25,26^

Bioorthogonal chemistry now enables the precise synthesis of protein therapeutics and drug conjugates, showing great potential in developing targeted therapies by facilitating site-specific attachment of therapeutic agents to enhance selectivity and reduce off-target effects.^27,28^ In the field of diagnostics, bioorthogonal reactions enable real-time imaging of biomolecules in living organisms, offering noninvasive approaches to disease monitoring.^29^ Metabolic labeling, a technique involving the incorporation of bioorthogonal groups into biomolecules, has provided a selective means for studying cellular processes such as protein synthesis and glycosylation.^18,19^

A detailed analysis of the concept of bioorthogonality in biochemical reactions reveals that it may present a significant challenge. While there are numerous impressive reactions that effectively yield the desired modifications, complications arise, particularly when employing several chemoselective reactions in parallel, e.g., in dual or multiple modifications.^30^ Here, one of the most important criteria of bioorthogonal chemistry, namely chemoselectivity, is often very challenging to achieve.^31,32^ This can result in unwanted reverse reactions and thus the loss of the covalent conjugation, decomposition of the chemical handle, or undesired side reactions between the two different (exogenous) groups. This scenario can be likened to a potent and “safe” drug in isolation, which may interact unpredictably when administered alongside a diverse array of other medications, leading to unforeseen side effects.

In this Perspective, we present a selected overview of prominent examples of side reactions in widely used bioorthogonal reactions. We would like to illustrate the scope and challenges of the field and provide a perspective for future chemical design strategies. While this selection offers insight into the topic, it should be noted that many additional examples are documented in the literature. This reflects the importance and broad application of bioorthogonal chemistry across diverse fields such as materials science, molecular imaging, and drug development.^1^

## Hercules at the Crossroads

**Figure 2:**
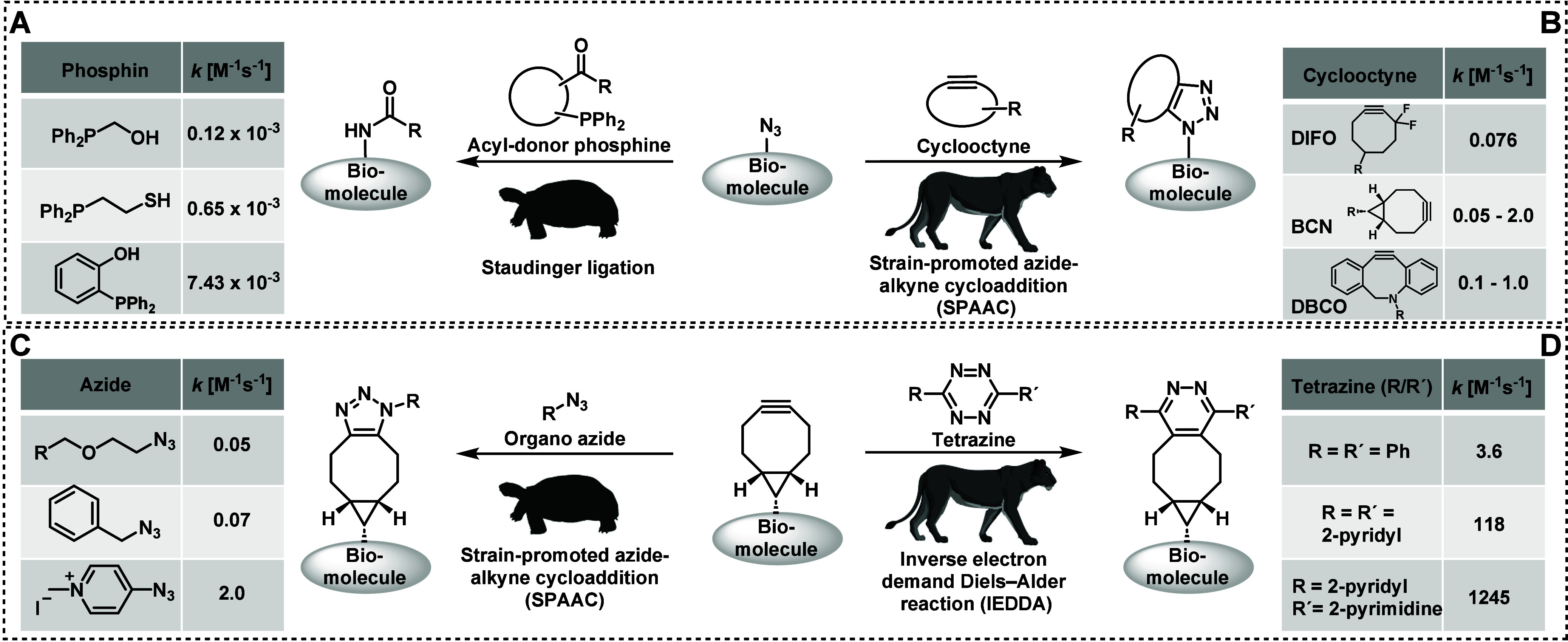
Overview of “slow” and “fast” bioconjugation reactions: A) Staudinger ligation from an organo azide and a phosphine with an acyl donor (slow).^33,34^ B) Strain-promoted azide–alkyne cycloaddition (SPAAC), starting from the same organo azide with a cyclooctyne (fast).^37^ C) Strain-promoted azide–alkyne cycloaddition (SPAAC), starting from a bicyclo[6.1.0]non-4-yne (BCN) (slow).^39^ D) Inverse electron-demand Diels–Alder (IEDDA) reaction, starting from the same BCN with a tetrazine (fast).^26^ Created by D. Schauenburg (2025) in BioRender (https://BioRender.com/u22t235).

In the realm of bioorthogonal chemistry, a single exogenous functional group can exhibit reactivity toward multiple reaction partners, depending on the specific conditions and the chemical environment. For instance, in the Staudinger ligation—a reaction between an organo azide and a phosphine (Figure 2A)^33,34^—the azide group undergoes nucleophilic attack by the phosphine, resulting in the formation of an amide bond and the release of nitrogen gas.^35^ However, what is often neglected is the fact that the Staudinger ligation can be considered as “slow”, with a second-order rate constant in the range of 10^–4^–10^–2^ M^–1^ s^–1^.^36^ Conversely, the widely applied strain-promoted azide–alkyne cycloaddition (SPAAC) reaction with a cyclooctyne proceeds via a (3+2) cycloaddition mechanism (Figure 2B).^37^ Here, the azide and cyclooctyne reactants undergo a highly selective and rapid cycloaddition reaction, facilitated by the inherent strain in the cyclooctyne ring. The second-order rate constant for this reaction is typically much higher (10^–2^–10^0^ M^–1^ s^–1^)^38,39^ than that of the Staudinger ligation, owing to the favorable reaction kinetics resulting from the strained alkyne. As a result, under kinetically controlled conditions, the SPAAC reaction is expected to predominate, leading to rapid formation of the desired cycloaddition product. However, under certain conditions—such as when cyclooctyne is present at lower concentrations or when reaction times are prolonged—the Staudinger ligation may also occur, yielding the corresponding amide product. Importantly, in applications such as cell surface labeling, where an excess of azides is present, the Staudinger ligation has been shown to proceed efficiently, as demonstrated in the pioneering work of Carolyn Bertozzi.^4^

The varying second-order rate constants and reaction mechanisms influence the relative product yields in chemoselective bioconjugation reactions, providing a way to control the outcome based on the respective reaction conditions. In the same way, cyclooctynes, such as bicyclo[6.1.0]non-4-yne (BCN), show dual reactivity, underscoring its potential utility in diverse chemical contexts, offering valuable options for selective and controlled transformations.^26^ BCN can undergo a (3+2) dipolar cycloaddition reaction with an azide (Figure 2C), the previously described SPAAC reaction, or engage in an inverse electron-demand Diels–Alder (IEDDA) reaction with a tetrazine (Figure 2D).^26^ In this example, the (3+2) cycloaddition is the “slow” reaction, with second-order rate constants in the range of 10^–3^–10^0^ M^–1^ s^–1^,^39^ whereas the tetrazine bioconjugation represents the “fast” reaction, which has second-order rate constants of 10^0^–10^3^ M^–1^ s^–1^.^26,40^

In addition to these two examples, there are other exogenous functional groups that can react with a wide variety of partners. In these reactions, the kinetics of the individual reactions control the expected product mixture. In the context of chemoselective bioconjugation reactions, careful consideration of the reaction kinetics is essential to select the appropriate conjugation method, aligning with the desired product outcome and experimental conditions. Understanding the intrinsic selectivity inherent to these reactions becomes particularly crucial when dealing with dual modifications or even more complex reaction conditions.^30^ This knowledge provides the basis for effective decision-making in experimental design, enabling researchers to navigate the complexities of bioconjugation reactions with precision and to achieve the desired outcomes.^2^

## Unexpected Side Reactions—Nothing Is Perfect

It is common for bioconjugation reactions to entail a two-step process.^41^ In the initial, “modification” step, a bifunctional small molecule is attached to a biomolecule as a chemical handle. In this phase, a large excess of the low-molecular-weight molecule is often used, facilitating straightforward purification methods such as size-exclusion chromatography, ultracentrifugation, or dialysis. In the second, “ligation” step, the two large molecules are connected through covalent bond formation. Unlike the “modification” step, ligation reactions are preferably conducted stoichiometrically to achieve precise bonding between often rare and valuable (bio)molecules. Especially in the first “modification” step, when a large excess of the chemical handle is used, unintended and unwanted side reactions can occur, which are discussed below.

**Figure 3:**
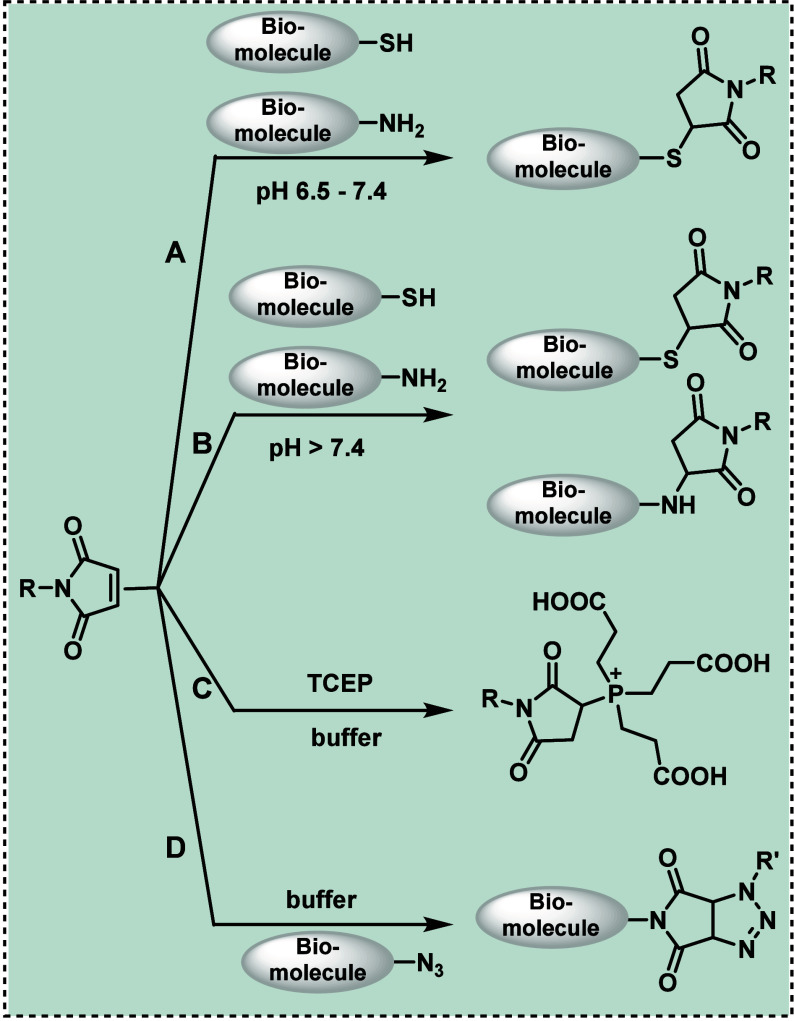
Reactions of maleimides: A) Site-selective modifications of thiols (e.g., cysteine side chains).^46^ B) Unselective modification of thiols and amines (e.g., cystine side chains (p*K*_a_ value 6.8 ± 2.7),^47^ N-terminus of peptides (p*K*_a_ value 7.7 ± 0.5),^47^ or lysine side chains (p*K*_a_ value 10.5 ± 1.1).^47^ C) Phospha-Michael addition of tris(2-carboxyethyl)phosphine (TCEP).^48^ D) Triazoline formation under (3+2) cycloaddition with an organo azide.^49^

A typical two-step reaction is the thiol-Michael addition reaction, which is used for the functionalization of thiol-containing biomolecules with maleimides. The roots of maleimide bioconjugation can be traced back^42,43^ more than 50 years, when chemists first recognized the potential of maleimides as electrophiles due to their capacity to react specifically with thiols, particularly cysteine residues in proteins (Figure 3A). This realization marked a pivotal moment, laying the foundation for a selective and efficient method for conjugating maleimides to biomolecules. The significance of thiol–maleimide chemistry is now apparent when considering, for example, the antibody–drug conjugate brentuximab vedotin, which received FDA approval for treating Hodgkin lymphoma.^44,45^ Here, the highly cytotoxic antimitotic drug monomethyl auristatin E is linked to a free cysteine residue of an antibody. Similarly, trastuzumab emtansine, which has been approved for the treatment of metastatic breast cancer, employs a maleimide moiety.^50−52^ In this case, the maleimide is conjugated to the antibody and reacts with a thiol group of the cytotoxic drug, thereby demonstrating the pivotal role played by thiol–maleimide chemistry in the context of these therapeutic applications.

It is nevertheless crucial to consider the potential retro-Michael addition as a back reaction, as it may ultimately result in cleavage and subsequent loss of the conjugate.^53^ Consequently, considerable efforts are devoted to the stabilization of maleimide conjugates, achieved through methods such as ring opening^54−56^ or *in situ* transcyclization, particularly when N-terminal cysteines are employed.^57^ If maleimide bioconjugation is carried out in alkaline conditions, when the pH of the reaction environment surpasses 7.5, there exists a potential hazard of nucleophilic addition reactions occurring with amines, e.g., the N-terminus of a peptide (p*K*_a_ value 7.7 ± 0.5)^47^ or lysine side chains (p*K*_a_ value 10.5 ± 1.1)^47^ (Figure 3B). These conditions serve to illustrate a pathway for potential off-target reactions. Conversely, we have recently demonstrated that, even in the presence of various Michael acceptors within a molecule, a thiol can be selectively conjugated to the maleimide under controlled pH conditions (at pH 6.0).

This is followed by two consecutive thiol additions to other Michael acceptors, achieved under stoichiometric control at pH 7.4.^58^ Apart from the challenges associated with site-selectivity in bioconjugation reactions, which are often due to unsuitable reaction conditions, maleimide-based bioconjugation can introduce an additional layer of complexity by inducing entirely different and often unexpected side reactions.

The thiol group, acting as a nucleophilic Michael addition partner, is often reduced *in situ* to prevent oxidation to the unreactive disulfide. Various reducing agents are employed for this purpose.^59^ When glutathione (GSH)^60^ or the more effective dithiothreitol (DTT)^61^ is utilized, it is evident that maleimides can react with these reagents, as they themselves contain thiol groups. Consequently, the maleimide can react with these reducing reagents if they are used in excess.^62^ However, what may not be immediately obvious is that the frequently used tris(2-carboxyethyl)phosphine (TCEP)^63^ also undergoes a phospha-Michael addition with maleimides (Figure 3C), leading to the formation of a phosphobetaine.^48,64,65^

The already introduced organo azides, such as azido homoalanine (AHA) or azido lysine, which can be easily incorporated into proteins through methods such as genetic encoding,^66,67^ serve as exogenous functional groups commonly employed in various chemical applications.^2^ Notably, their relatively small size contributes to their versatility, allowing for facile incorporation into diverse molecular contexts.^68^ This functional group exhibits remarkable stability under both basic and acidic reaction conditions, making it well-suited for a range of synthetic procedures. It proves particularly valuable in the Staudinger ligation^33^ and copper(I)-catalyzed azide–alkyne cycloaddition (CuAAC)^17,69^ reactions. In these reactions, the organo azide participates in the formation of a native amide^70^ or triazole,^71,72^ a molecular motif that is considered as an isoster of the amide bond.^73^ Less attention has been paid to the unintended (side) reaction of a maleimide and an azide, which involves a dipolar cycloaddition (Figure 3D).^26^ The diene-like system in the maleimide serves as an electron-rich environment, while the triple bond in the azide creates an electron-deficient site.^49^ The initial stage of the reaction involves the nucleophilic attack of the azide’s nitrogen lone pair on the electron-deficient β-carbon of the maleimide, which initiates a (3+2) cycloaddition, yielding a stable, five-membered triazoline ring comprising two carbon atoms from the maleimide and three nitrogen atoms contributed by the azide.^49,65^ This cycloaddition is not only highly efficient but also selective, giving rise to a covalently linked product, but it has a relatively slow reaction rate (10^–7^–10^–5^ M s^–1^).^49^

The stability of the triazoline ring further ensures robustness of the formed product. For this reason, it is not surprising that there are actually no bifunctional molecules commercially available that have both an azide and a maleimide, as triazoline formation would occur during isolation and storage. To overcome these limitations, so-called “maleimide–azide kits” are commercially available.^74^ They consist of an alkyl azide and amine, which are often separated from each other by some PEG units, and a maleimide NHS-ester. Since triazoline formation has a rather slow second-order rate constant, it is possible to prepare the maleimide–azide linker *in situ*. This bifunctional linker then has to be used immediately for the modification of thiols (e.g., cystines), which enables the generation of an azide-modified biomolecule.

**Figure 4:**
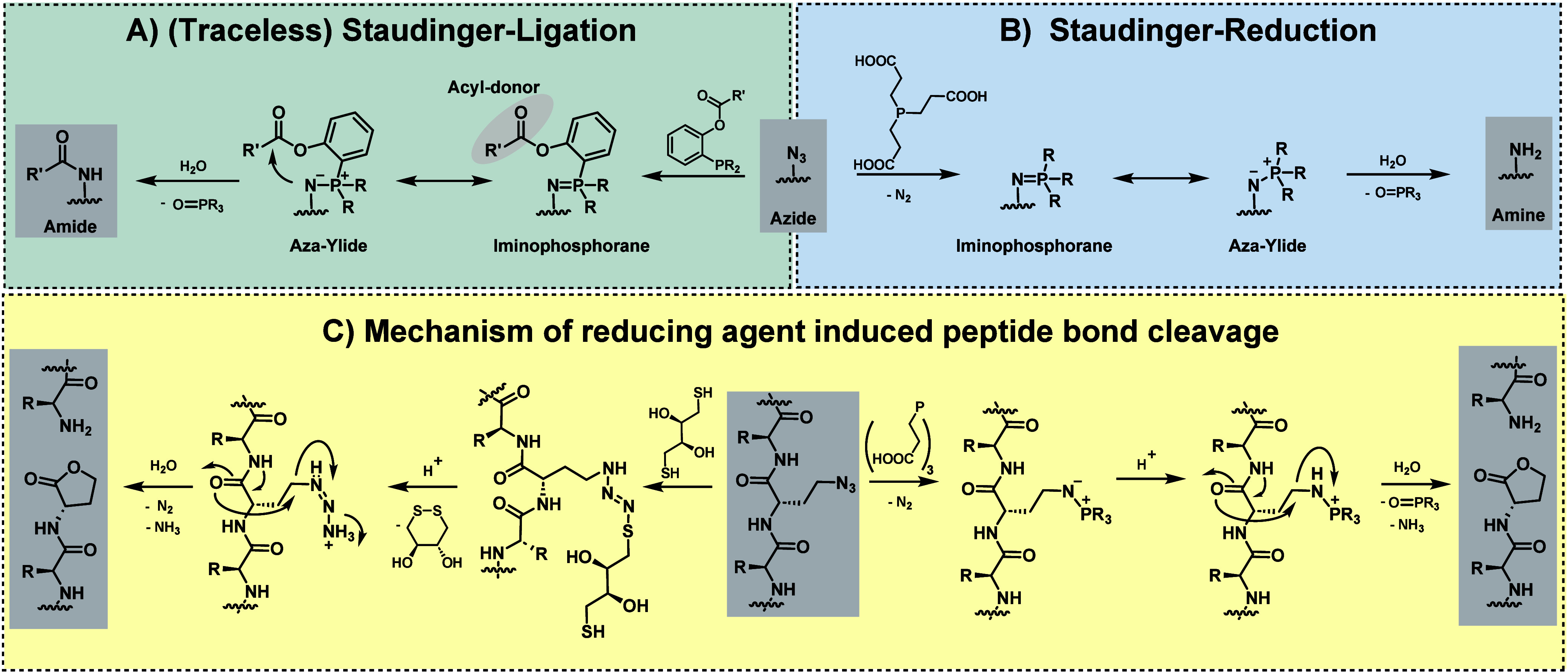
Reactions of organo azides: A) Amide-forming (traceless) Staudinger ligation.^77^ B) Amine-forming Staudinger reduction.^97^ C) Peptide bond cleavage after an azido homoalanine residue using dithiothreitol (DTT) or tris(2-carboxyethyl)phosphine (TCEP).^78^

The reaction of an organo azide with a phosphine represents a pivotal step in he Staudinger ligation (Figure 4A). Its origins can be traced back to the Staudinger reduction, a well-established chemical transformation first reported in 1919 by Hermann Staudinger.^75,76^ If no acyl donor is present, as in the Staudinger ligation, the resulting azide–ylide undergoes hydrolysis in an aqueous buffer to afford the corresponding primary amine (Figure 4B).^33,77^ This may be very useful in organic chemistry for amine masking; however, it presents a challenge for bioconjugation reactions. In a scenario where biomolecules are modified with azides and subsequent disulfide reduction is performed using TCEP, unintended azide reduction leading to amines will occur, necessitating careful consideration in bioconjugation strategies. The cleavage of peptide bonds by TCEP or DTT is a further consequence of the reaction, as exemplified by the cleavage of azido homoalanine residues. In the Staudinger reduction of azido homoalanine, a reactive triazene intermediate is formed, subsequently leading to the generation of a cyclic imidoester (Figure 4C).^78^ Ultimately, this cascade of reactions results in the cleavage of the peptide bond, yielding a C-terminal homoserine lactone and an N-terminal amine. This chemical cleavage of amide (peptide) bonds is exceptionally mild and occurs within a broad pH range, typically spanning from pH 5 to 7. As a concurrent side reaction, the aza–ylide pathway is also observed, leading to the hydrolysis of the intermediate to form the amine, specifically 2,4-diaminobutyric acid (DAB). Should this reaction be desired, it can be a very useful tool for the targeted degradation of biopolymers. However, if unwanted, the Staudinger reduction can result in the loss of the azide (e.g., azido lysine) or even breakage of the peptide chain (e.g., azido homoalanine).

## Metal-Catalyzed Bioconjugation Reactions

In addition to the bioconjugation reactions discussed so far, where molecules A and B react to form product C through covalent bond formation, we will now discuss side products formed during catalyzed reactions. A catalyst is defined as a chemical agent capable of accelerating chemical reactions by introducing new reaction pathways.^79^ It remains unchanged throughout the reaction, neither consumed nor altering the thermodynamic equilibrium position. Today, a plethora of transition-metal-catalyzed bioconjugation reactions are documented in literature.^80−82^ Examples include palladium-catalyzed cross-couplings,^83,84^ oxidative addition complexes specifically targeting thiols,^85,86^ ruthenium-catalyzed olefin metathesis,^87−89^ rhodium-catalyzed tyrosine functionalization,^90−92^ and gold-catalyzed amide bond formation.^93,94^ However, these represent only a fraction of the potential applications of metal-catalyzed bioconjugation reactions, which have been applied *in vitro* and *in vivo*.^2^ As an illustrative example, we will consider one of the most prevalent “click” reactions, the previously mentioned CuAAC reaction, a variant of the azide–alkyne Huisgen cycloaddition.^95,96^

Initially, this reaction was perceived as very slow due to the absence of ring strain in the terminal alkyne. Huisgen conducted the experiments primarily at elevated temperatures. Subsequently in 2002, Sharpless^72^ and Meldal^98^ independently discovered that copper(I) can serve as a catalyst. Thanks to its remarkable selectivity and practicality, CuAAC has found widespread applications beyond chemistry, extending into fields like biology and materials science.^99−101^ Nevertheless, even this “good” reaction carries the potential for unexpected side reactions.^102^

**Figure 5:**
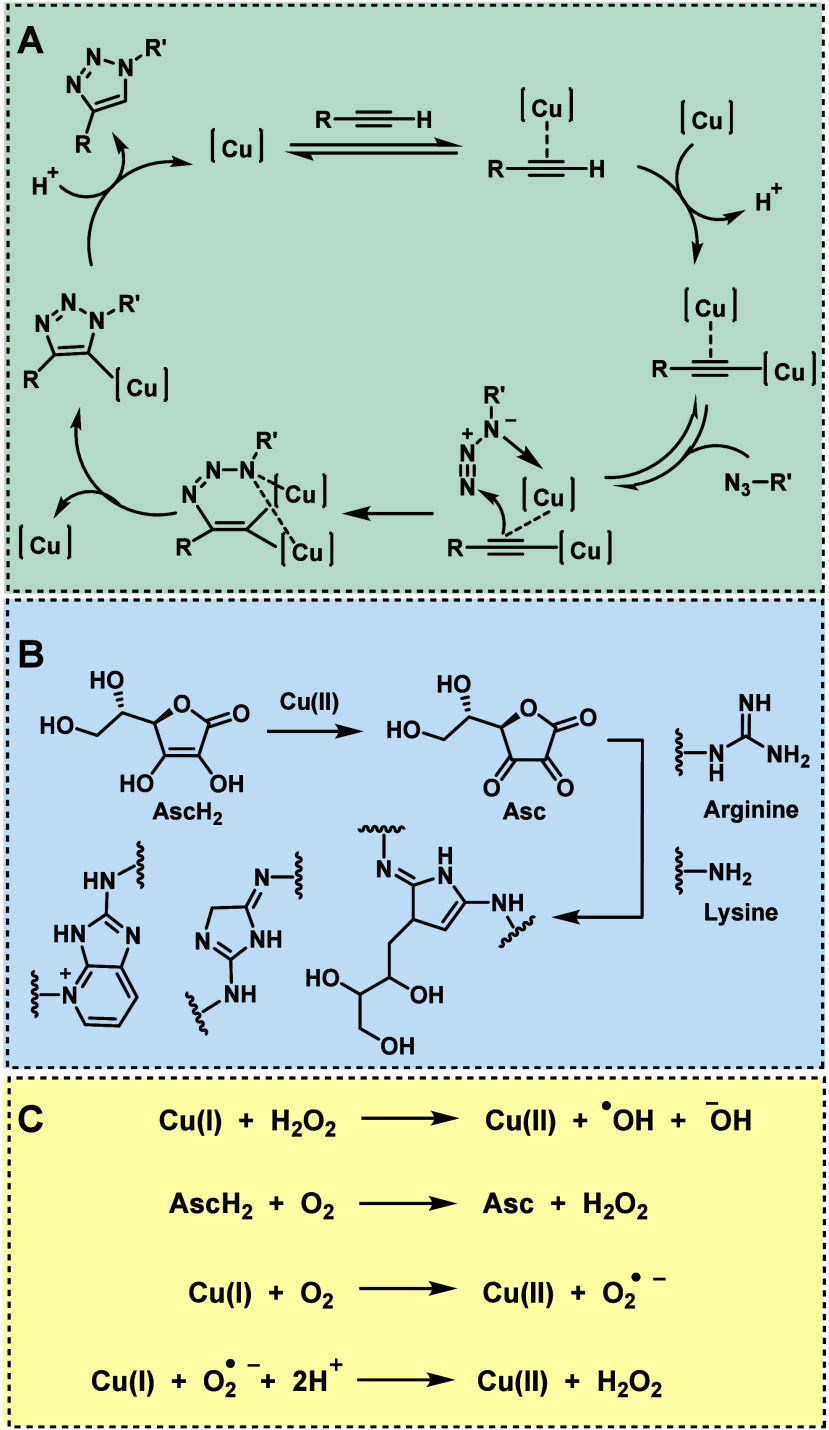
Copper(I)-catalyzed azide–alkyne cycloaddition (CuAAC): A) Catalytic cycle.^101^ B) Reduction of copper(II) to catalytically active copper(I) by ascorbic acid (AscH_2_) and possible protein cross-linking of lysine or arginine side chains with the resulting dehydroascorbate (Asc).^101^ C) Copper-mediated generation of reactive oxygen species that can react with side chains of amino acids in proteins, including tyrosine, methionine, cysteine, and tryptophan, leading to potential modifications and functional changes in protein structure and activity.^101,103^

The mechanism of this triazole-forming reaction entails coordination of the copper(I) catalyst with the terminal alkyne, thereby forming a copper acetylide complex (Figure 5A). The CuAAC reaction is distinguished by its mechanism, which involves the transient formation of a highly reactive coordination complex of alkynes and azides on a Cu(I) cluster stabilized by ligands and reactants.^101^ This templated organization of reactants is crucial for the reaction’s success, enabling it to proceed efficiently at low concentrations where two copper atoms scramble in the transition state. The mechanism, influenced by the reaction environment, has been studied extensively, with current evidence suggesting that the C-2 of the alkyne acts as an electrophile in the transition state, supported by observed kinetics and structural analyses.

Due to the instability of the catalytically active copper(I) in aqueous solutions, which tends to disproportionate into copper(II) and copper(0), copper(II) is frequently employed in the reaction, which is subsequently reduced *in situ* to copper(I). As a mild reducing agent, ascorbic acid (AscH_2_) or its salts are commonly utilized for this purpose (Figure 5B). The ascorbate-based reducing agent utilized in these reactions can pose a risk due to the electrophilic nature of its oxidized form, dehydroascorbate (Asc). Protein cross-linking of lysine, arginine, and cysteine side chains by Asc^104,105^ and the oxidation of amino acid side chains (e.g., histidine)^106^ have been reported in literature. Due to the inherent instability of catalytically active Cu(I) under physiological conditions, oxidative stress and cytotoxicity linked to CuAAC are associated with the capability of Cu(I) to generate reactive oxygen species (ROS) from O_2_ (Figure 5C).^107,108^ The oxidation to Cu(II), facilitated by either O_2_ or H_2_O_2_ (via a Fenton processes), promotes the production of superoxide or hydroxyl radicals, respectively.^109,110^ The presence of ROS can disrupt the structural and functional integrity of biomolecules, leading to DNA base oxidation, lipid peroxidation, and protein carbonylation—processes that have been observed under CuAAC conditions.^111^ Cu(I)-stabilizing ligands are essential in Cu-catalyzed click reactions, preventing Cu(I) oxidation to Cu(II) and suppressing the formation of ROS that could lead to side reactions.^112^ By coordinating to Cu(I), these ligands enhance reaction efficiency and selectivity. Commonly used ligands include tris(benzyltriazolylmethyl)amine (TBTA), which improves Cu(I) solubility, bathophenanthroline, known for its strong chelation and oxidative stability, and *N,N,N′,N″,N′′*-pentamethyldiethylenetriamine (PMDTA), which accelerates reaction rates while maintaining catalyst stability.^112^ The choice of ligand depends on reaction conditions and substrate compatibility, ensuring optimal performance in CuAAC reactions.^113−115^ In addition to metal ions such as copper, ROS can also be generated by light exposure, leading to the formation of highly reactive intermediates.

## Reactive Intermediates in Photochemistry

**Figure 6:**
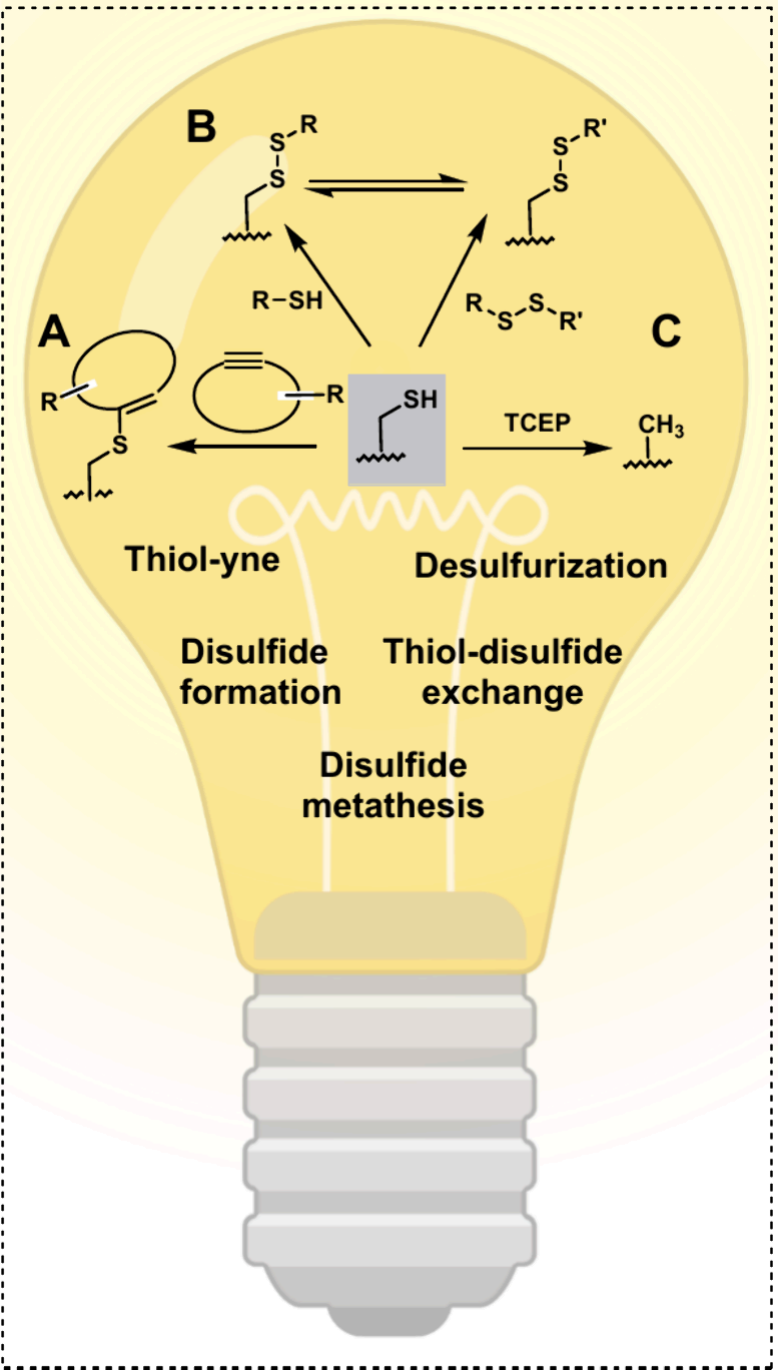
UV-light-induced reaction of thiol groups: A) Thiol–yne reaction with a (strained) alkyne.^116^ B) Disulfide formation, thiol–disulfide exchange, and disulfide metathesis.^117,118^ C) Thiol desulfurization.^119^ Created by D. Schauenburg (2025) in BioRender (https://BioRender.com/u22t235).

The intricate interplay between photochemical reactions and bioconjugation processes can give rise to a multitude of side reactions that may potentially influence the desired biomolecular modifications. This phenomenon presents both opportunities for further research and challenges that must be addressed. One notable example involves the interaction between excited states or radicals generated during photochemical reactions and thiol-containing biomolecules, where irradiation can induce undesired side reactions that compromise the thiol functionality and affect the outcome of the modification. As previously outlined, the Michael addition, which typically involves the addition of a nucleophile to the electrophilic β-carbon of an α,β-unsaturated carbonyl compound, represents one pathway of bioconjugation reactions.^120^ These reactions are typically catalyzed by bases or acids and proceed through the formation of an enolate or enamine intermediate. In contrast, a thiol–ene or thiol–yne reaction involves the addition of a thiol to an alkene or alkyne, respectively, in the presence of a photoinitiator or a radical initiator.^121−124^ Thiol–yne reactions are characterized by their high efficiency, selectivity, and tolerance to various functional groups. The mechanism involves the initiation of a radical chain reaction by the photoinitiator or radical initiator, followed by addition of the thiol to the alkyne to form a carbon–sulfur bond (Figure 6A).^116^ In studies of photoinitiated thiol addition reactions involving various alkynes, it has been demonstrated that cyclooctynes exhibit the highest reactivity.^116^ This finding poses a significant challenge in selecting functional groups, particularly strained alkynes, for SPAAC reactions. The rapid reactivity of cyclooctynes can complicate the desired sequence of chemical transformations in synthetic pathways. Moreover, *in vitro* experiments have provided insights into the mechanisms underlying azide-independent protein labeling with cyclooctynes.^125^ Among the reactive functionalities investigated, cysteine thiols facilitate the covalent attachment of cyclooctynes to proteins. This discovery underscores the necessity for a comprehensive understanding of the specific reactivity profiles of functional groups in bioconjugation strategies, particularly in the context of site-selective labeling and biomolecule modification. Thiols play a crucial role in dynamic covalent chemistry, particularly in biological systems, where they contribute to essential processes such as protein folding through reversible disulfide bond formation and polyketide biosynthesis via thioester exchange. Their ability to form and break covalent bonds under physiological conditions makes them highly relevant to bioorthogonal chemistry, offering versatile strategies for selective biomolecular modifications.^126,127^ Under ultraviolet light, thiols exhibit dynamic reactive behavior, participating in processes such as oxidation to disulfides, thiol–disulfide exchange, and disulfide metathesis, driven by radical intermediates (Figure 6B).^117,118,128^

Apart from facilitating thiol additions to form new covalent bonds, photoirradiation can induce the cleavage of sulfur–carbon bonds. This phenomenon, termed photoinduced desulfurization, occurs upon exposure of thiol-containing molecules to UV light in the presence of mild reducing agents, such as TCEP (Figure 6C).^119^ This transformation is often used in native chemical ligation (NCL), a key method in chemical protein synthesis.^129^ This ligation is a chemoselective method for connecting two peptide fragments through the reaction between a C-terminal thioester group and an N-terminal cysteine residue, resulting in the formation of a native peptide bond.^130,131^

Another potential side reaction involves the interaction of photochemically generated radicals with amino groups, such as primary amines in lysine residues or amidyl radicals from amide bonds.^132^ These radicals can participate in cross-linking reactions or undergo further chemical transformations, leading to undesired modifications of nitrogen-containing groups (e.g., unwanted thiol oxidation).^117^ Excited states, particularly those derived from UV-absorbing species, can sensitize molecular oxygen to produce ROS, which can induce side reactions, as explained in the previous section; the generation of ROS by UV radiation is one of the mechanisms involving wavelengths shorter than 400 nm.^133^

Moreover, reactions involving nucleic acids can lead to DNA or RNA damage when photochemically generated species interact with nucleobases.^134−136^ For example, radicals generated during photochemical reactions can abstract hydrogen atoms from pyrimidine nucleobases, resulting in the formation of DNA or RNA radicals, which can lead to strand breaks or modifications; specifically, RNA nucleobase radicals directly cause strand breaks, while DNA nucleobase peroxyl radicals produce tandem lesions.^137^

To mitigate these side reactions, researchers often employ radical scavengers or antioxidants to minimize the impact of ROS.^142−144^ Careful selection of reaction conditions, such as the choice of wavelength for photoexcitation and the concentration of reactants, can help to optimize bioconjugation reactions while minimizing undesired modifications caused by reactive intermediates originating from the photochemical processes.

**Figure 7:**
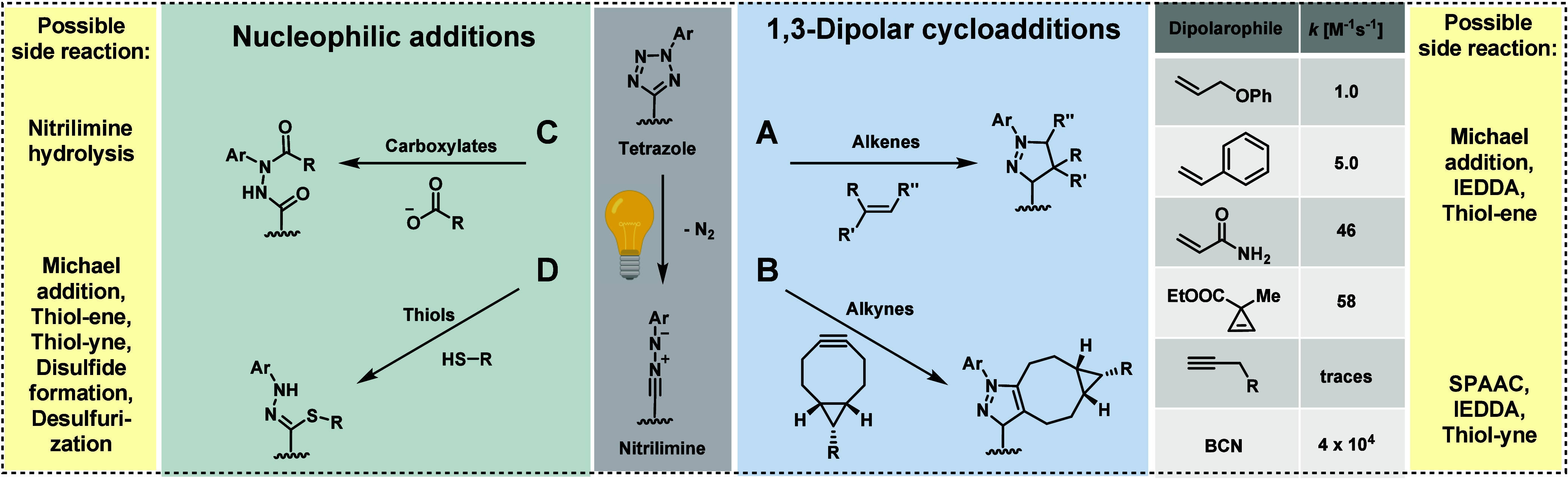
Tetrazole photoclick chemistry: A) 1,3-Dipolar cycloaddition with alkenes.^138^ B) 1,3-Dipolar cycloaddition with (strained) alkynes.^139^ C) Nucleophilic addition of carboxylates.^140^ D) Nucleophilic addition of thiols.^141^ Possible side reactions occur with the employed reaction partners. This diagram illustrates the various unintended reactions that may occur alongside the main reaction, highlighting the potential interactions between the different reactants and their byproducts. Created by D. Schauenburg (2025) in BioRender (https://BioRender.com/z55l791).

In addition to the numerous transformations (either desired or undesired) of endogenous functional groups that are orchestrated by UV light, there exists another fascinating realm of chemical reactions known as “photoclick” reactions, which are often employed in bioconjugation strategies.^145,146^ In theory these modifications offer precise control and selectivity, making them valuable tools for various applications, from biochemistry to materials science.^147,148^ For instance, one prominent photoclick reaction involves the UV-induced (around 350 nm) dimerization of coumarins, leading to the formation of cyclobutane adducts.^149^ However, these versatile reactions can reveal off-site selectivity, as some exogenous chemical entities have the potential to cross-react, as described below. To delve into the intricacies and the complexity of UV-mediated bioconjugation reactions, we will first focus on a specific type known as tetrazole photoclick chemistry.^150^ Tetrazoles, fundamental heterocyclic compounds, consist of a five-membered ring comprising four nitrogens and one carbon atom.^151^ They can undergo transformations to highly reactive intermediates under UV irradiation (typically between 250 and 400 nm), depending on the substituents attached.^145^ The reactive intermediate that emerges from tetrazole under UV light has been identified as nitrilimine (Figure 7). Due to its electronic structure, it can undergo 1,3-dipolar cycloadditions with substituted alkenes (Figure 7A).^138^ When employing relatively unreactive alkenes, the reactions exhibit a moderate second-order rate constant (e.g., allyl ether: 1 M^–1^ s^–1^, styrene: 5 M^–1^ s^–1^).^145,152^ However, with more electron-deficient alkenes like maleimides,^153^ acrylates,^138^ or acrylamides,^145^ the reactions accelerate. Conversely, these compounds also serve as proficient Michael acceptors, capable of reacting with nucleophiles such as thiols.^154^

A good balance between reactivity and stability is found in cyclopropenes, which undergo tetrazole conjugation with a second-order rate constant ranging from 10 to 60 M^–1^ s^–1^.^26,155^ In contrast, terminal or aliphatically substituted alkynes exhibit slow reactivity, making them unsuitable for bioconjugation. However, the reaction rate accelerates when electron-withdrawing groups, such as esters (acetylenedicarboxylate), are introduced; similar to the azide–alkyne Huisgen cycloaddition reaction, the tetrazole photoclick reaction significantly enhances reaction kinetics through ring strain. Consequently, cyclooctyne reacts much faster than linear alkynes, extending to BCN (Figure 7B). “Superfast tetrazole–BCN cycloaddition” with second-order rate constants up to 10^4^ M^–1^ s^–1^ has been recently reported.^139^

Besides its reactivity toward alkenes and alkynes, the reactive nitrilium intermediate exhibits a propensity to react with various nucleophiles such as thiols, carboxylates, and hydroxyl and amino groups, challenging its bioorthogonality.^141^ Whereas carboxylates yield the bis-acetylated hydrazines^140^ (Figure 7C), stronger nucleophiles such as thiols attack on the nitrile carbon and give the hydrazonothioate (Figure 7D).^141,156^ Additionally, the hydrolysis of nitrileimines in water further complicates their use in bioconjugation applications.

The final category of phototransformations employed in bioconjugation reactions, as outlined in this Perspective, encompasses photoremovable protecting groups. These are also referred to as photocleavable or photolabile protecting groups that can be reversibly attached to functional groups, which temporarily masks their reactivity or properties.^157−159^ These groups can selectively be cleaved or removed upon exposure to light of a specific wavelength (usually between 250 and 500 nm), which restores the original functionality.^160^ This light-induced cleavage allows for precise spatial and temporal control over the activation or deactivation of biomolecules, making photolabile protecting groups valuable tools in various fields such as drug delivery,^161^ material science,^162^ and bioconjugation chemistry.^163^ The kinetics of deprotection typically involves a photolysis process where absorption of photons by the photoremovable protecting group induces cleavage, liberating the functional group with high efficiency and minimal side reactions, thus enabling precise spatiotemporal control over biomolecular interactions.^164^

These reactions are especially effective in biomolecular applications because they adhere to a zero-order rate law and can function under very dilute conditions (μM concentrations and below). This characteristic implies that the reaction rate remains constant and independent of substrate concentration, enabling precise control over the reaction kinetics.^165,166^

Bode and co-workers convincingly illustrated how chemically synthesized analogues of Interleukin-4 (IL-4),^167,168^ bearing a single photocaged amino acid side chain (Gln116 residue), could be activated by UV light, effectively suppressing neutrophils in an inflammation model *in vivo*.^169,170^

Recently, our group has demonstrated that intermediates generated during the photodeprotection of functional groups can serve as oxidizing agents for thiols.^171^ Specifically, the 2-nitroveratryloxycarbonyl (Nvoc) group exhibits absorption of ultraviolet light at 365 nm, leading to the formation of a zwitterionic excited state. Subsequent cleavage of the N=O nitro bond results in the release of CO_2_.^157^ The resulting aromatic N=O nitroso group possesses the capability to oxidize thiols into disulfides.^172^ Combining both processes, our group has proposed that thiol-containing molecules protected with the Nvoc group could undergo a self-induced transformation into disulfides *in situ.* This tandem reaction capitalizes on the distinctive photochemical properties of the Nvoc group to enable controlled and sequential thiol oxidation.^171^

## Dyes in Bioorthogonal Chemistry

While not directly related to bioconjugation *per se*, the discussion of fluorescent dyes is pertinent due to their widespread use, and it seems crucial to address possible side reactions. Fluorescent dyes, ubiquitous tools in various scientific applications, are susceptible to degradation mechanisms that can compromise their utility and reliability.^3,173^

One well-known challenge is the photodegradation of fluorophores induced by exposure to light, including natural sunlight or UV irradiation.^174^ This process is particularly relevant in fluorescence microscopy and imaging applications, where dyes are exposed to intense light sources.^175^ The energy absorbed by the fluorophores during excitation can lead to the generation of reactive species or the cleavage of chemical bonds within the dye molecule, resulting in reduced fluorescence intensity or complete loss of fluorescence.^176^ Photobleaching is particularly problematic in long-term imaging experiments, where prolonged exposure may compromise the accuracy of fluorescence data.^177^ However, it is important to consider that these compounds are exposed to UV radiation during photocatalytic reactions involving biomolecules. Light exposure, especially in the presence of oxygen, can trigger photooxidation reactions that may cause irreversible damage to the dye molecules, thereby compromising their fluorescence properties.^178,179^

As described before, numerous dye molecules exhibit characteristics of good Michael acceptors, owing to their extensive π-systems. This property renders them capable of functioning as electrophiles and reacting with a variety of nucleophiles (endogenous and exogenous). The large π-systems present in these dye molecules provide ample electron-deficient regions that attract nucleophilic species, facilitating the formation of covalent bonds through Michael addition reactions.^180−182^

**Figure 8:**
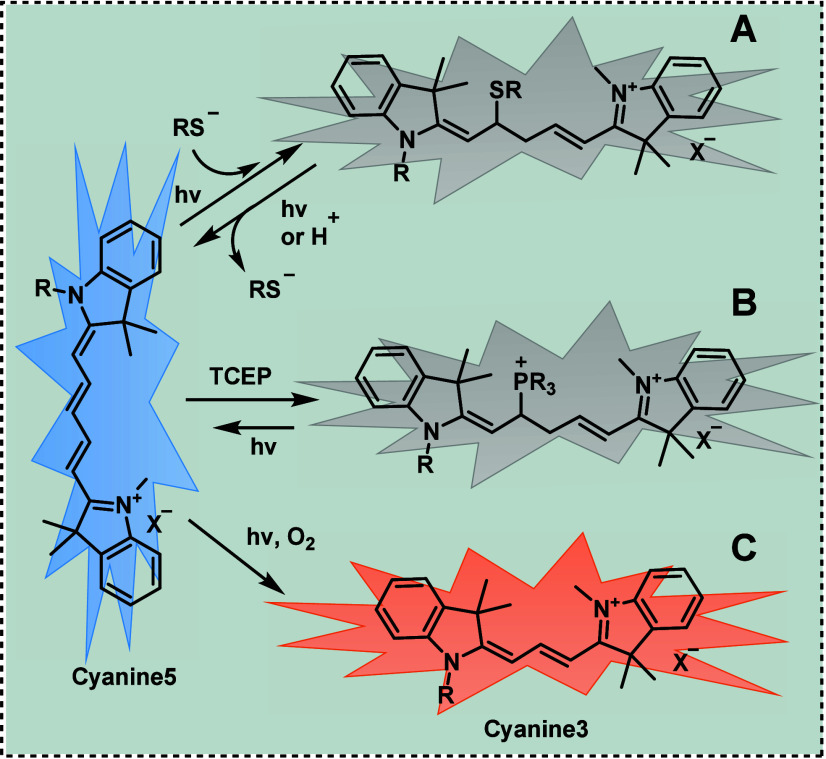
Reactions of cyanine5 (Cy5) dyes: A) Thiol-induced photoswitching of cyanine dyes.^185^ B) Phosphine quenching of cyanine dyes.^186^ C) Photoconversion of cyanine5 to cyanine3.^184^

It has been proposed that thiyl radicals may engage in reactions with alkenes or other conjugated systems such as polymethine groups in cyanine (Cy) dyes, to generate adducts.^183,184^ This suggests the potential involvement of radical intermediates in the reaction, where electron transfer from the thiol anion to the cyanine precedes covalent bond formation and loss of molecule fluorescence (Figure 8A).^185^

Utilizing single-molecule imaging and mass spectrometry, the mechanism of photoswitching of cyanine dyes has been investigated. These analyses revealed that the conversion to the dark state is contingent upon pH and thiol concentration, yielding a cyanine–thiol adduct as the resultant product. Vaughan et al. have demonstrated that exogenous phosphines like TCEP can participate in a 1,4-addition to the polymethine bridge of Cy5, resulting in the formation of covalent adducts that lead to fluorescence quenching (Figure 8B).^186^ Exposure to UV light dissociates the adduct, restoring the dye to its fluorescent state.

These two chosen examples for on–off modulation of fluorophores by reversible addition to the π-system carry considerable potential benefits, especially in super-resolution imaging applications. However, without recognition of this phenomenon, the absence of fluorescence might lead to misinterpretation of the results. This issue is particularly critical in complex systems such as living cells or organisms, where precise, interdisciplinary analyses are challenging to conduct.

Moreover, extending beyond the dye on–off switch, the conversion of Cy5 to another dye, specifically Cy3, via a photochemical process involving the unique excision of C_2_H_2_ from the polymethine chain has been observed in living cells (Figure 8C).^184^ Through the identification of photoproducts and intermediates, a chemical reaction mechanism for the transformation of Cy5 to Cy3 has been proposed. This process is initiated by photooxidation-induced cleavage of the polymethine chain of Cy5, followed by condensation of the resulting carbonyl products and Fischer’s base to produce Cy3. Importantly, this photoconversion reaction is also applicable to other far-red-emitting indocarbocyanine dyes like AF647. Given that even ambient light can induce the photoconversion of far-red organic fluorophores, the presence of yellow-emissive dyes is inevitable in single-molecule studies and super-resolution imaging of living cells.

## Summary and Outlook

Bioorthogonal chemistry and click chemistry have revolutionized multiple scientific disciplines, which was recognized by the 2022 Nobel Prize in Chemistry. These reactions are essential for the precise modification of biomolecules, driving advancements in fields such as synthetic biology, protein therapeutics, and vaccine development. However, significant challenges remain, including reaction reversibility, unintended side reactions, and complexities introduced by catalysts and photochemical activation. Addressing these challenges requires the continuous development of innovative strategies to enhance chemoselectivity, efficiency, and stability, particularly in complex biological environments. A deeper understanding of potential side reactions is crucial, especially in the context of living cells, where analytical limitations are obvious.

Despite the transformative impact of bioorthogonal chemistry, there is still significant room for further development. Future efforts should focus on designing bioconjugation reagents and reaction systems with improved stability, selectivity, and biocompatibility in complex and living environments. A significant challenge persists in the execution of bioorthogonal reactions under physiological conditions without interfering with endogenous biological processes. Reagents that exhibit enhanced resistance to hydrolysis, oxidation, and metabolic degradation would significantly improve their applicability for long-term studies of biological systems. Additionally, increasing the specificity toward particular biomolecules, such as proteins, glycans, or nucleic acids, could enable highly targeted modifications and functionalization. Performing bioorthogonal chemistry within living cells or whole organisms presents unique hurdles, including competing biological reactions, limited reagent accessibility, and potential cytotoxicity. Expanding the repertoire of bioorthogonal reactions to operate efficiently in intracellular environments is therefore essential. Strategies involving the design of membrane-permeable reagents, catalyst-free reactions, and photoactivatable bioorthogonal tools hold great promise for improving reaction control in living systems. Furthermore, developing real-time monitoring and regulation methods for bioorthogonal reactions *in vivo* will be critical for achieving spatial and temporal precision. An exciting but underexplored frontier is the execution of multiple bioorthogonal reactions in parallel, allowing for double, triple, or even higher-order functionalization. Such approaches would enable simultaneous labeling or modification of multiple biomolecules within the same system, facilitating advanced studies of complex cellular networks and biochemical pathways. Nevertheless, considerable challenges persist, including the assurance of orthogonality between reaction partners, the maintenance of reagent compatibility, and the optimization of reaction kinetics. Engineering distinct reactivity profiles and minimizing cross-reactivity are key to achieving multifunctional bioorthogonal strategies. Interestingly, side reactions—often considered undesirable—could offer valuable new opportunities for reaction discovery. For example, the controlled generation of radicals under bioorthogonal conditions remains largely unexplored but may serve as a foundation for novel chemistries. Future research could focus on elucidating the mechanisms and consequences of these side reactions within biological systems. By harnessing these processes, scientists could uncover new pathways for targeted functionalization, biomolecule activation, and therapeutic applications.

In conclusion, achieving precise control and a thorough understanding of bioorthogonal reactions and their byproducts is crucial for unlocking their full potential in increasingly sophisticated biological systems. The future of bioorthogonal chemistry lies in overcoming these challenges to enable high chemoselectivity and access to more complex reaction networks with spatial and temporal control. Ultimately, a deeper mechanistic insight into both desired and unintended reactions will pave the way for groundbreaking applications, ranging from cellular imaging and synthetic biology to therapeutic interventions.

## References

[ref1] Scinto S. L.; Bilodeau D. A.; Hincapie R.; Lee W.; Nguyen S. S.; Xu M.; am Ende C. W.; Finn M. G.; Lang K.; Lin Q.; Pezacki J. P.; Prescher J. A.; Robillard M. S.; Fox J. M. Bioorthogonal Chemistry. Nat. Rev. Methods Prim. 2021, 1 (1), 30 10.1038/s43586-021-00028-z. PMC8469592 34585143

[ref2] Schauenburg D.; Weil T. Chemical Reactions in Living Systems. Adv. Sci. 2024, 11, 2303396 10.1002/advs.202303396. PMC10885656 37679060

[ref3] Bird R. E.; Lemmel S. A.; Yu X.; Zhou Q. A. Bioorthogonal Chemistry and Its Applications. Bioconjugate Chem. 2021, 32 (12), 2457–2479. 10.1021/acs.bioconjchem.1c00461. 34846126

[ref4] Sletten E. M.; Bertozzi C. R. Bioorthogonal Chemistry: Fishing for Selectivity in a Sea of Functionality. Angew. Chem., Int. Ed. 2009, 48 (38), 6974–6998. 10.1002/anie.200900942. PMC2864149 19714693

[ref5] Lang K.; Chin J. W. Bioorthogonal Reactions for Labeling Proteins. ACS Chem. Biol. 2014, 9 (1), 16–20. 10.1021/cb4009292. 24432752

[ref6] Liu H.; Wang Y.; Zhou X. Labeling and Sequencing Nucleic Acid Modifications Using Bio-Orthogonal Tools. RSC Chem. Biol. 2022, 3 (8), 994–1007. 10.1039/D2CB00087C. 35975003 PMC9347354

[ref7] Merkel M.; Peewasan K.; Arndt S.; Ploschik D.; Wagenknecht H. Copper-free Postsynthetic Labeling of Nucleic Acids by Means of Bioorthogonal Reactions. ChemBioChem 2015, 16 (11), 1541–1553. 10.1002/cbic.201500199. 26063100

[ref8] El-Sagheer A. H.; Brown T. Click Chemistry with DNA. Chem. Soc. Rev. 2010, 39 (4), 1388–1405. 10.1039/b901971p. 20309492

[ref9] Mikutis S.; Gu M.; Sendinc E.; Hazemi M. E.; Kiely-Collins H.; Aspris D.; Vassiliou G. S.; Shi Y.; Tzelepis K.; Bernardes G. J. L. MeCLICK-Seq, a Substrate-Hijacking and RNA Degradation Strategy for the Study of RNA Methylation. ACS Cent. Sci. 2020, 6 (12), 2196–2208. 10.1021/acscentsci.0c01094. 33376781 PMC7760485

[ref10] Saxon E.; Bertozzi C. R. Cell Surface Engineering by a Modified Staudinger Reaction. Science 2000, 287 (5460), 2007–2010. 10.1126/science.287.5460.2007. 10720325

[ref11] Wang Y.; Hu Q. Bio-Orthogonal Chemistry in Cell Engineering. Adv. NanoBiomed. Res. 2023, 3 (3), 2200128 10.1002/anbr.202200128.

[ref12] Abbina S.; Siren E. M. J.; Moon H.; Kizhakkedathu J. N. Surface Engineering for Cell-Based Therapies: Techniques for Manipulating Mammalian Cell Surfaces. ACS Biomater. Sci. Eng. 2018, 4 (11), 3658–3677. 10.1021/acsbiomaterials.7b00514. 33429616

[ref13] Ramil C. P.; Lin Q. Bioorthogonal Chemistry: Strategies and Recent Developments. Chem. Commun. 2013, 49 (94), 11007–11022. 10.1039/c3cc44272a. PMC3847904 24145483

[ref14] Wright M. H. Chemical Biology Tools for Protein Labelling: Insights into Cell–Cell Communication. Biochem. J. 2023, 480 (18), 1445–1457. 10.1042/BCJ20220309. 37732646 PMC10586760

[ref15] Hou J.; Liu X.; Shen J.; Zhao G.; Wang P. G. The Impact of Click Chemistry in Medicinal Chemistry. Expert Opin. Drug Discovery 2012, 7 (6), 489–501. 10.1517/17460441.2012.682725. 22607210

[ref16] Moses J. E.; Moorhouse A. D. The Growing Applications of Click Chemistry. Chem. Soc. Rev. 2007, 36 (8), 1249–1262. 10.1039/B613014N. 17619685

[ref17] Kolb H. C.; Finn M. G.; Sharpless K. B. Click Chemistry: Diverse Chemical Function from a Few Good Reactions. Angew. Chem., Int. Ed. 2001, 40 (11), 2004–2021. 10.1002/1521-3773(20010601)40:11<2004::AID-ANIE2004>3.0.CO;2-5. 11433435

[ref18] Prescher J. A.; Bertozzi C. R. Chemistry in Living Systems. Nat. Chem. Biol. 2005, 1 (1), 13–21. 10.1038/nchembio0605-13. 16407987

[ref19] Baskin J. M.; Bertozzi C. R. Bioorthogonal Click Chemistry: Covalent Labeling in Living Systems. QSAR Comb. Sci. 2007, 26 (11–12), 1211–1219. 10.1002/qsar.200740086.

[ref20] Sletten E. M.; Bertozzi C. R. From Mechanism to Mouse: A Tale of Two Bioorthogonal Reactions. Acc. Chem. Res. 2011, 44 (9), 666–676. 10.1021/ar200148z. 21838330 PMC3184615

[ref21] Bertozzi C. R. A Decade of Bioorthogonal Chemistry. Acc. Chem. Res. 2011, 44 (9), 651–653. 10.1021/ar200193f. 21928847 PMC4408923

[ref22] Patterson D. M.; Nazarova L. A.; Prescher J. A. Finding the Right (Bioorthogonal) Chemistry. ACS Chem. Biol. 2014, 9 (3), 592–605. 10.1021/cb400828a. 24437719

[ref23] Saito F.; Noda H.; Bode J. W. Critical Evaluation and Rate Constants of Chemoselective Ligation Reactions for Stoichiometric Conjugations in Water. ACS Chem. Biol. 2015, 10 (4), 1026–1033. 10.1021/cb5006728. 25572124

[ref24] Brimble M. A.; Ackermann L.; Li Y.-M.; Raj M. Chemoselective Methods for Labeling and Modification of Peptides and Proteins. Org. Lett. 2023, 25, 6605–6606. 10.1021/acs.orglett.3c02630. 37711046

[ref25] Dirksen A.; Dawson P. E. Rapid Oxime and Hydrazone Ligations with Aromatic Aldehydes for Biomolecular Labeling. Bioconjugate Chem. 2008, 19 (12), 2543–2548. 10.1021/bc800310p. PMC2761707 19053314

[ref26] Oliveira B. L.; Guo Z.; Bernardes G. J. L. Inverse Electron Demand Diels–Alder Reactions in Chemical Biology. Chem. Soc. Rev. 2017, 46 (16), 4895–4950. 10.1039/C7CS00184C. 28660957

[ref27] Taiariol L.; Chaix C.; Farre C.; Moreau E. Click and Bioorthogonal Chemistry: The Future of Active Targeting of Nanoparticles for Nanomedicines?. Chem. Rev. 2022, 122 (1), 340–384. 10.1021/acs.chemrev.1c00484. 34705429

[ref28] Yi W.; Xiao P.; Liu X.; Zhao Z.; Sun X.; Wang J.; Zhou L.; Wang G.; Cao H.; Wang D.; Li Y. Recent Advances in Developing Active Targeting and Multi-Functional Drug Delivery Systems via Bioorthogonal Chemistry. Signal Transduct. Target. Ther. 2022, 7 (1), 386 10.1038/s41392-022-01250-1. 36460660 PMC9716178

[ref29] Kenry; Liu B. Bio-Orthogonal Click Chemistry for in Vivo Bioimaging. Trends Chem. 2019, 1 (8), 763–778. 10.1016/j.trechm.2019.08.003.

[ref30] Xu L.; Kuan S. L.; Weil T. Contemporary Approaches for Site-Selective Dual Functionalization of Proteins. Angew. Chem., Int. Ed. 2021, 60 (25), 13757–13777. 10.1002/anie.202012034. PMC8248073 33258535

[ref31] Afagh N. A.; Yudin A. K. Chemoselectivity and the Curious Reactivity Preferences of Functional Groups. Angew. Chem., Int. Ed. 2010, 49 (2), 262–310. 10.1002/anie.200901317. 20014082

[ref32] Devaraj N. K. The Future of Bioorthogonal Chemistry. ACS Cent. Sci. 2018, 4 (8), 952–959. 10.1021/acscentsci.8b00251. 30159392 PMC6107859

[ref33] Bednarek C.; Wehl I.; Jung N.; Schepers U.; Bräse S. The Staudinger Ligation. Chem. Rev. 2020, 120 (10), 4301–4354. 10.1021/acs.chemrev.9b00665. 32356973

[ref34] Saxon E.; Armstrong J. I.; Bertozzi C. R. A Traceless” Staudinger Ligation for the Chemoselective Synthesis of Amide Bonds. Org. Lett. 2000, 2 (14), 2141–2143. 10.1021/ol006054v. 10891251

[ref35] Lin F. L.; Hoyt H. M.; van Halbeek H.; Bergman R. G.; Bertozzi C. R. Mechanistic Investigation of the Staudinger Ligation. J. Am. Chem. Soc. 2005, 127 (8), 2686–2695. 10.1021/ja044461m. 15725026

[ref36] Soellner M. B.; Nilsson B. L.; Raines R. T. Reaction Mechanism and Kinetics of the Traceless Staudinger Ligation. J. Am. Chem. Soc. 2006, 128 (27), 8820–8828. 10.1021/ja060484k. 16819875

[ref37] Agard N. J.; Prescher J. A.; Bertozzi C. R. A Strain-Promoted [3+2] Azide–Alkyne Cycloaddition for Covalent Modification of Biomolecules in Living Systems. J. Am. Chem. Soc. 2004, 126 (46), 15046–15047. 10.1021/ja044996f. 15547999

[ref38] Forshaw S.; Parker J. S.; Scott W. T.; Knighton R. C.; Tiwari N.; Oladeji S. M.; Stevens A. C.; Chew Y. M.; Reber J.; Clarkson G. J.; Balasubramanian M. K.; Wills M. Increasing the Versatility of the Biphenyl-Fused-Dioxacyclodecyne Class of Strained Alkynes. Org. Biomol. Chem. 2024, 22 (3), 590–605. 10.1039/D3OB01712E. 38131271 PMC10792613

[ref39] Dommerholt J.; Van Rooijen O.; Borrmann A.; Guerra C. F.; Bickelhaupt F. M.; Van Delft F. L. Highly Accelerated Inverse Electron-Demand Cycloaddition of Electron-Deficient Azides with Aliphatic Cyclooctynes. Nat. Commun. 2014, 5 (1), 5378 10.1038/ncomms6378. 25382411

[ref40] Wang D.; Chen W.; Zheng Y.; Dai C.; Wang K.; Ke B.; Wang B. 3, 6-Substituted-1, 2, 4, 5-Tetrazines: Tuning Reaction Rates for Staged Labeling Applications. Org. Biomol. Chem. 2014, 12 (23), 3950–3955. 10.1039/c4ob00280f. 24806890 PMC4149905

[ref41] King T. A.; Pérez L. R.; Flitsch S. L. Application of Biocatalysis for Protein Bioconjugation. Comprehensive Chirality 2024, 389–437. 10.1016/B978-0-32-390644-9.00122-0.

[ref42] Renault K.; Fredy J. W.; Renard P.-Y.; Sabot C. Covalent Modification of Biomolecules through Maleimide-Based Labeling Strategies. Bioconjugate Chem. 2018, 29 (8), 2497–2513. 10.1021/acs.bioconjchem.8b00252. 29954169

[ref43] Northrop B. H.; Frayne S. H.; Choudhary U. Thiol–Maleimide “Click” Chemistry: Evaluating the Influence of Solvent, Initiator, and Thiol on the Reaction Mechanism, Kinetics, and Selectivity. Polym. Chem. 2015, 6 (18), 3415–3430. 10.1039/C5PY00168D.

[ref44] Katz J.; Janik J. E.; Younes A. Brentuximab Vedotin (SGN-35). Clin. cancer Res. 2011, 17 (20), 6428–6436. 10.1158/1078-0432.CCR-11-0488. 22003070

[ref45] Younes A.; Yasothan U.; Kirkpatrick P. Brentuximab Vedotin. Nat. Rev. Drug Discovery 2012, 11 (1), 19 10.1038/nrd3629. 22212672

[ref46] Stenzel M. H. Bioconjugation Using Thiols: Old Chemistry Rediscovered to Connect Polymers with Nature’s Building Blocks. ACS Macro Lett. 2013, 2 (1), 14–18. 10.1021/mz3005814. 35581832

[ref47] Grimsley G. R.; Scholtz J. M.; Pace C. N. A Summary of the Measured PK Values of the Ionizable Groups in Folded Proteins. Protein Sci. 2009, 18 (1), 247–251. 10.1002/pro.19. 19177368 PMC2708032

[ref48] Enders D.; Saint-Dizier A.; Lannou M.; Lenzen A. The Phospha-Michael Addition in Organic Synthesis. Eur. J. Org. Chem. 2006, 2006 (1), 29–49. 10.1002/ejoc.200500593.

[ref49] Sinclair A. J.; Del Amo V.; Philp D. Structure–Reactivity Relationships in a Recognition Mediated [3+ 2] Dipolar Cycloaddition Reaction. Org. Biomol. Chem. 2009, 7 (16), 3308–3318. 10.1039/b908072d. 19641790

[ref50] LoRusso P. M.; Weiss D.; Guardino E.; Girish S.; Sliwkowski M. X. Trastuzumab Emtansine: A Unique Antibody-Drug Conjugate in Development for Human Epidermal Growth Factor Receptor 2–Positive Cancer. Clin. Cancer Res. 2011, 17 (20), 6437–6447. 10.1158/1078-0432.CCR-11-0762. 22003071

[ref51] García-Alonso S.; Ocaña A.; Pandiella A. Trastuzumab Emtansine: Mechanisms of Action and Resistance, Clinical Progress, and Beyond. Trends in cancer 2020, 6 (2), 130–146. 10.1016/j.trecan.2019.12.010. 32061303

[ref52] Barok M.; Joensuu H.; Isola J. Trastuzumab Emtansine: Mechanisms of Action and Drug Resistance. Breast Cancer Res. 2014, 16, 209 10.1186/bcr3621. 24887180 PMC4058749

[ref53] Szijj P. A.; Bahou C.; Chudasama V. Minireview: Addressing the Retro-Michael Instability of Maleimide Bioconjugates. Drug Discovery Today Technol. 2018, 30, 27–34. 10.1016/j.ddtec.2018.07.002. 30553517

[ref54] Fontaine S. D.; Reid R.; Robinson L.; Ashley G. W.; Santi D. V. Long-Term Stabilization of Maleimide–Thiol Conjugates. Bioconjugate Chem. 2015, 26 (1), 145–152. 10.1021/bc5005262. 25494821

[ref55] Lyon R. P; Setter J. R; Bovee T. D; Doronina S. O; Hunter J. H; Anderson M. E; Balasubramanian C. L; Duniho S. M; Leiske C. I; Li F.; Senter P. D Self-Hydrolyzing Maleimides Improve the Stability and Pharmacological Properties of Antibody-Drug Conjugates. Nat. Biotechnol. 2014, 32 (10), 1059–1062. 10.1038/nbt.2968. 25194818

[ref56] Vasco A. V.; Taylor R. J.; Méndez Y.; Bernardes G. J. On-Demand Thio-Succinimide Hydrolysis for the Assembly of Stable Protein–Protein Conjugates. J. Am. Chem. Soc. 2024, 146 (30), 20709–20719. 10.1021/jacs.4c03721. 39012647 PMC11295205

[ref57] Lahnsteiner M.; Kastner A.; Mayr J.; Roller A.; Keppler B. K.; Kowol C. R. Improving the Stability of Maleimide–Thiol Conjugation for Drug Targeting. Chem.—Eur. J. 2020, 26 (68), 15867–15870. 10.1002/chem.202003951. 32871016 PMC7756610

[ref58] Schauenburg D.; Zech F.; Heck A. J.; von Maltitz P.; Harms M.; Führer S.; Alleva N.; Münch J.; Kuan S. L.; Weil T.; Kirchhoff F. Peptide Bispecifics Inhibiting HIV-1 Infection by an Orthogonal Chemical and Supramolecular Strategy. Bioconjugate Chem. 2023, 34 (9), 1645–1652. 10.1021/acs.bioconjchem.3c00314. PMC10515486 37665137

[ref59] Mthembu S. N.; Sharma A.; Albericio F.; de la Torre B. G. Breaking a Couple: Disulfide Reducing Agents. ChemBioChem 2020, 21 (14), 1947–1954. 10.1002/cbic.202000092. 32196882

[ref60] Meister A.; Anderson M. E. Glutathione. Annu. Rev. Biochem. 1983, 52 (1), 711–760. 10.1146/annurev.bi.52.070183.003431. 6137189

[ref61] Cleland W. W. Dithiothreitol, a New Protective Reagent for SH Groups. Biochemistry 1964, 3 (4), 480–482. 10.1021/bi00892a002. 14192894

[ref62] Henkel M.; Röckendorf N.; Frey A. Selective and Efficient Cysteine Conjugation by Maleimides in the Presence of Phosphine Reductants. Bioconjugate Chem. 2016, 27 (10), 2260–2265. 10.1021/acs.bioconjchem.6b00371. 27631603

[ref63] Burns J. A.; Butler J. C.; Moran J.; Whitesides G. M. Selective Reduction of Disulfides by Tris (2-Carboxyethyl) Phosphine. J. Org. Chem. 1991, 56 (8), 2648–2650. 10.1021/jo00008a014.

[ref64] Lee Y.; Kurra Y.; Liu W. R. Phospha-Michael Addition as a New Click Reaction for Protein Functionalization. ChemBioChem 2016, 17 (6), 456–461. 10.1002/cbic.201500697. 26756316 PMC5059841

[ref65] Kantner T.; Alkhawaja B.; Watts A. G. In Situ Quenching of Trialkylphosphine Reducing Agents Using Water-Soluble PEG-Azides Improves Maleimide Conjugation to Proteins. ACS Omega 2017, 2 (9), 5785–5791. 10.1021/acsomega.7b01094. 30023752 PMC6044941

[ref66] Plass T.; Milles S.; Koehler C.; Schultz C.; Lemke E. A. Genetically Encoded Copper-Free Click Chemistry. Angew. Chem., Int. Ed. 2011, 50 (17), 3878 10.1002/anie.201008178. PMC3210829 21433234

[ref67] Nguyen D. P.; Lusic H.; Neumann H.; Kapadnis P. B.; Deiters A.; Chin J. W. Genetic Encoding and Labeling of Aliphatic Azides and Alkynes in Recombinant Proteins via a Pyrrolysyl-TRNA Synthetase/TRNACUA Pair and Click Chemistry. J. Am. Chem. Soc. 2009, 131 (25), 8720–8721. 10.1021/ja900553w. 19514718

[ref68] Kiick K. L.; Saxon E.; Tirrell D. A.; Bertozzi C. R. Incorporation of Azides into Recombinant Proteins for Chemoselective Modification by the Staudinger Ligation. Proc. Natl. Acad. Sci. U. S. A. 2002, 99 (1), 19–24. 10.1073/pnas.012583299. 11752401 PMC117506

[ref69] Wu P.; Feldman A. K.; Nugent A. K.; Hawker C. J.; Scheel A.; Voit B.; Pyun J.; Fréchet J. M. J.; Sharpless K. B.; Fokin V. V. Efficiency and Fidelity in a Click-Chemistry Route to Triazole Dendrimers by the Copper(I)-Catalyzed Ligation of Azides and Alkynes. Angew. Chem., Int. Ed. 2004, 43 (30), 3928–3932. 10.1002/anie.200454078. 15274216

[ref70] Pattabiraman V. R.; Bode J. W. Rethinking Amide Bond Synthesis. Nature 2011, 480 (7378), 471–479. 10.1038/nature10702. 22193101

[ref71] Valverde I. E.; Mindt T. L. 1, 2, 3-Triazoles as Amide-Bond Surrogates in Peptidomimetics. Chim. Int. J. Chem. 2013, 67 (4), 262–266. 10.2533/chimia.2013.262. 23967702

[ref72] Rostovtsev V. V.; Green L. G.; Fokin V. V.; Sharpless K. B. A Stepwise Huisgen Cycloaddition Process: Copper(I)-Catalyzed Regioselective “Ligation” of Azides and Terminal Alkynes. Angew. Chem., Int. Ed. 2002, 41 (14), 2596–2599. 10.1002/1521-3773(20020715)41:14<2596::AID-ANIE2596>3.0.CO;2-4. 12203546

[ref73] Bonandi E.; Christodoulou M. S.; Fumagalli G.; Perdicchia D.; Rastelli G.; Passarella D. The 1, 2, 3-Triazole Ring as a Bioisostere in Medicinal Chemistry. Drug Discovery Today 2017, 22 (10), 1572–1581. 10.1016/j.drudis.2017.05.014. 28676407

[ref74] Zoppelt J. M. Smart Protein-Based Therapeutics. Thesis, Johannes Gutenberg-Universität Mainz, 2023.

[ref75] Staudinger H.; Meyer J. Über Neue Organische Phosphorverbindungen III. Phosphinmethylenderivate Und Phosphinimine. Helv. Chim. Acta 1919, 2 (1), 635–646. 10.1002/hlca.19190020164.

[ref76] Liu S.; Edgar K. J. Staudinger Reactions for Selective Functionalization of Polysaccharides: A Review. Biomacromolecules 2015, 16 (9), 2556–2571. 10.1021/acs.biomac.5b00855. 26245299

[ref77] Köhn M.; Breinbauer R. The Staudinger Ligation—a Gift to Chemical Biology. Angew. Chem., Int. Ed. 2004, 43 (24), 3106–3116. 10.1002/anie.200401744. 15199557

[ref78] Back J. W.; David O.; Kramer G.; Masson G.; Kasper P. T.; de Koning L. J.; de Jong L.; van Maarseveen J. H.; de Koster C. G. Mild and Chemoselective Peptide-Bond Cleavage of Peptides and Proteins at Azido Homoalanine. Angew. Chem., Int. Ed. 2005, 44 (48), 7946–7950. 10.1002/anie.200502431. 16281315

[ref79] Richardson J. T. Principles of Catalyst Development; Springer, 2013.

[ref80] Rodríguez J.; Martínez-Calvo M. Transition-Metal-Mediated Modification of Biomolecules. Chem.—Eur. J. 2020, 26 (44), 9792–9813. 10.1002/chem.202001287. 32602145

[ref81] Antos J. M.; Francis M. B. Transition Metal Catalyzed Methods for Site-Selective Protein Modification. Curr. Opin. Chem. Biol. 2006, 10 (3), 253–262. 10.1016/j.cbpa.2006.04.009. 16698310

[ref82] Patra M.; Gasser G. Organometallic Compounds: An Opportunity for Chemical Biology?. ChemBioChem 2012, 13 (9), 1232–1252. 10.1002/cbic.201200159. 22619182

[ref83] Jbara M.; Maity S. K.; Brik A. Palladium in the Chemical Synthesis and Modification of Proteins. Angew. Chem., Int. Ed. 2017, 56 (36), 10644–10655. 10.1002/anie.201702370. 28383786

[ref84] Dibowski H.; Schmidtchen F. P. Bioconjugation of Peptides by Palladium-Catalyzed C– C Cross-Coupling in Water. Angew. Chem., Int. Ed. 1998, 37 (4), 476–478. 10.1002/(SICI)1521-3773(19980302)37:4<476::AID-ANIE476>3.0.CO;2-2. 29711173

[ref85] Kubota K.; Dai P.; Pentelute B. L.; Buchwald S. L. Palladium Oxidative Addition Complexes for Peptide and Protein Cross-Linking. J. Am. Chem. Soc. 2018, 140 (8), 3128–3133. 10.1021/jacs.8b00172. 29406701 PMC5831526

[ref86] Rojas A. J.; Wolfe J. M.; Dhanjee H. H.; Buslov I.; Truex N. L.; Liu R. Y.; Massefski W.; Pentelute B. L.; Buchwald S. L. Palladium–Peptide Oxidative Addition Complexes for Bioconjugation. Chem. Sci. 2022, 13 (40), 11891–11895. 10.1039/D2SC04074C. 36320916 PMC9580489

[ref87] Vinogradova E. V. Organometallic Chemical Biology: An Organometallic Approach to Bioconjugation. Pure Appl. Chem. 2017, 89 (11), 1619–1640. 10.1515/pac-2017-0207.

[ref88] Gutiérrez-González A.; Marcos-Atanes D.; Cool L. G.; López F.; Mascareñas J. L. Ruthenium-Catalyzed Intermolecular Alkene–Alkyne Couplings in Biologically Relevant Media. Chem. Sci. 2023, 14 (23), 6408–6413. 10.1039/D3SC01254A. 37325130 PMC10266458

[ref89] Lin Y. A.; Chalker J. M.; Davis B. G. Olefin Metathesis for Site-selective Protein Modification. ChemBioChem 2009, 10 (6), 959–969. 10.1002/cbic.200900002. 19343741

[ref90] Ohata J.; Miller M. K.; Mountain C. M.; Vohidov F.; Ball Z. T. A Three-component Organometallic Tyrosine Bioconjugation. Angew. Chem., Int. Ed. 2018, 57 (11), 2827–2830. 10.1002/anie.201711868. 29356233

[ref91] Alvarez Dorta D.; Deniaud D.; Mével M.; Gouin S. G. Tyrosine Conjugation Methods for Protein Labelling. Chem.—Eur. J. 2020, 26 (63), 14257–14269. 10.1002/chem.202001992. 32538529

[ref92] Ohata J.; Ball Z. T. Rhodium at the Chemistry–Biology Interface. Dalton Trans. 2018, 47 (42), 14855–14860. 10.1039/C8DT03032D. 30234200

[ref93] Lo V. K.-Y.; Chan A. O.-Y.; Che C.-M. Gold and Silver Catalysis: From Organic Transformation to Bioconjugation. Org. Biomol. Chem. 2015, 13 (24), 6667–6680. 10.1039/C5OB00407A. 25997423

[ref94] Tsubokura K.; Vong K. K. H.; Pradipta A. R.; Ogura A.; Urano S.; Tahara T.; Nozaki S.; Onoe H.; Nakao Y.; Sibgatullina R.; Kurbangalieva A.; Watanabe Y.; Tanaka K. In Vivo Gold Complex Catalysis within Live Mice. Angew. Chem., Int. Ed. 2017, 56 (13), 3579–3584. 10.1002/anie.201610273. 28198119

[ref95] Huisgen R. Centenary Lecture–1,3-Dipolar Cycloadditions. Royal Soc Chemistry Thomas Graham House, Science Park, Milton Rd, Cambridge, U.K., 1961.

[ref96] Huisgen R. 1.3-Dipolare Cycloadditionen Rückschau und Ausblick. Angew. Chem. 1963, 75 (13), 604–637. 10.1002/ange.19630751304.

[ref97] Leffler J. E.; Temple R. D. Staudinger Reaction between Triarylphosphines and Azides. Mechanism. J. Am. Chem. Soc. 1967, 89 (20), 5235–5246. 10.1021/ja00996a027.

[ref98] Tornøe C. W.; Christensen C.; Meldal M. Peptidotriazoles on Solid Phase: [1,2,3]-Triazoles by Regiospecific Copper(I)-Catalyzed 1,3-Dipolar Cycloadditions of Terminal Alkynes to Azides. J. Org. Chem. 2002, 67 (9), 3057–3064. 10.1021/jo011148j. 11975567

[ref99] Agrahari A. K.; Bose P.; Jaiswal M. K.; Rajkhowa S.; Singh A. S.; Hotha S.; Mishra N.; Tiwari V. K. Cu (I)-Catalyzed Click Chemistry in Glycoscience and Their Diverse Applications. Chem. Rev. 2021, 121 (13), 7638–7956. 10.1021/acs.chemrev.0c00920. 34165284

[ref100] Haldón E.; Nicasio M. C.; Pérez P. J. Copper-Catalysed Azide–Alkyne Cycloadditions (CuAAC): An Update. Org. Biomol. Chem. 2015, 13 (37), 9528–9550. 10.1039/C5OB01457C. 26284434

[ref101] Meldal M.; Diness F. Recent Fascinating Aspects of the CuAAC Click Reaction. Trends Chem. 2020, 2 (6), 569–584. 10.1016/j.trechm.2020.03.007.

[ref102] McKay C. S.; Finn M. G. Click Chemistry in Complex Mixtures: Bioorthogonal Bioconjugation. Chem. Biol. 2014, 21 (9), 1075–1101. 10.1016/j.chembiol.2014.09.002. 25237856 PMC4331201

[ref103] Andrés C. M. C.; Pérez de la Lastra J. M.; Andrés Juan C.; Plou F. J.; Pérez-Lebeña E. Impact of Reactive Species on Amino Acids—Biological Relevance in Proteins and Induced Pathologies. Int. J. Mol. Sci. 2022, 23 (22), 14049 10.3390/ijms232214049. 36430532 PMC9692786

[ref104] Reihl O.; Lederer M. O.; Schwack W. Characterization and Detection of Lysine–Arginine Cross-Links Derived from Dehydroascorbic Acid. Carbohydr. Res. 2004, 339 (3), 483–491. 10.1016/j.carres.2003.12.004. 15013385

[ref105] Kay P.; Wagner J. R.; Gagnon H.; Day R.; Klarskov K. Modification of Peptide and Protein Cysteine Thiol Groups by Conjugation with a Degradation Product of Ascorbate. Chem. Res. Toxicol. 2013, 26 (9), 1333–1339. 10.1021/tx400061e. 23865753

[ref106] Hong V.; Presolski S. I.; Ma C.; Finn M. â G. Analysis and Optimization of Copper-Catalyzed Azide–Alkyne Cycloaddition for Bioconjugation. Angew. Chem., Int. Ed. 2009, 48 (52), 9879 10.1002/anie.200905087. PMC3410708 19943299

[ref107] Brewer G. J. Risks of Copper and Iron Toxicity during Aging in Humans. Chem. Res. Toxicol. 2010, 23 (2), 319–326. 10.1021/tx900338d. 19968254

[ref108] Brotherton W. S.; Michaels H. A.; Simmons J. T.; Clark R. J.; Dalal N. S.; Zhu L. Apparent Copper (II)-Accelerated Azide– Alkyne Cycloaddition. Org. Lett. 2009, 11 (21), 4954–4957. 10.1021/ol9021113. 19810690

[ref109] Sutton H. C.; Winterbourn C. C. On the Participation of Higher Oxidation States of Iron and Copper in Fenton Reactions. Free Radic. Biol. Med. 1989, 6 (1), 53–60. 10.1016/0891-5849(89)90160-3. 2536343

[ref110] Pham A. N.; Xing G.; Miller C. J.; Waite T. D. Fenton-like Copper Redox Chemistry Revisited: Hydrogen Peroxide and Superoxide Mediation of Copper-Catalyzed Oxidant Production. J. Catal. 2013, 301, 54–64. 10.1016/j.jcat.2013.01.025.

[ref111] Juan C. A.; Pérez de la Lastra J. M.; Plou F. J.; Pérez-Lebeña E. The Chemistry of Reactive Oxygen Species (ROS) Revisited: Outlining Their Role in Biological Macromolecules (DNA, Lipids and Proteins) and Induced Pathologies. Int. J. Mol. Sci. 2021, 22 (9), 4642 10.3390/ijms22094642. 33924958 PMC8125527

[ref112] Meldal M.; Tornøe C. W. Cu-Catalyzed Azide–Alkyne Cycloaddition. Chem. Rev. 2008, 108 (8), 2952–3015. 10.1021/cr0783479. 18698735

[ref113] Binder W. H.; Kluger C. Azide/Alkyne-“Click” Reactions: Applications in Material Science and Organic Synthesis. Curr. Org. Chem. 2006, 10 (14), 1791–1815. 10.2174/138527206778249838.

[ref114] Bevilacqua V.; King M.; Chaumontet M.; Nothisen M.; Gabillet S.; Buisson D.; Puente C.; Wagner A.; Taran F. Copper-chelating Azides for Efficient Click Conjugation Reactions in Complex Media. Angew. Chem. 2014, 126 (23), 5982–5986. 10.1002/ange.201310671. 24788475

[ref115] Struthers H.; Mindt T. L.; Schibli R. Metal Chelating Systems Synthesized Using the Copper (I) Catalyzed Azide-Alkyne Cycloaddition. Dalton Trans. 2010, 39 (3), 675–696. 10.1039/B912608B. 20066208

[ref116] Fairbanks B. D.; Sims E. A.; Anseth K. S.; Bowman C. N. Reaction Rates and Mechanisms for Radical, Photoinitated Addition of Thiols to Alkynes, and Implications for Thiol– Yne Photopolymerizations and Click Reactions. Macromolecules 2010, 43 (9), 4113–4119. 10.1021/ma1002968.

[ref117] Li L.; Feng W.; Welle A.; Levkin P. A. UV-Induced Disulfide Formation and Reduction for Dynamic Photopatterning. Angew. Chem. 2016, 128 (44), 13969–13973. 10.1002/ange.201607276. 27699955

[ref118] Lechner V. M.; Nappi M.; Deneny P. J.; Folliet S.; Chu J. C. K.; Gaunt M. J. Visible-Light-Mediated Modification and Manipulation of Biomacromolecules. Chem. Rev. 2022, 122 (2), 1752–1829. 10.1021/acs.chemrev.1c00357. 34546740

[ref119] Ma Y.; Deng J.; Gu J.; Jiang D.; Lv K.; Ye X.; Yao Q. Recent Progress in Photoinduced Direct Desulfurization of Thiols. Org. Biomol. Chem. 2023, 21, 7873–7879. 10.1039/D3OB01274C. 37750040

[ref120] Mather B. D.; Viswanathan K.; Miller K. M.; Long T. E. Michael Addition Reactions in Macromolecular Design for Emerging Technologies. Prog. Polym. Sci. 2006, 31 (5), 487–531. 10.1016/j.progpolymsci.2006.03.001.

[ref121] Lowe A. B. Thiol-Ene “Click” Reactions and Recent Applications in Polymer and Materials Synthesis. Polym. Chem. 2010, 1 (1), 17–36. 10.1039/B9PY00216B.

[ref122] Hoyle C. E.; Bowman C. N. Thiol–Ene Click Chemistry. Angew. Chem., Int. Ed. 2010, 49 (9), 1540–1573. 10.1002/anie.200903924. 20166107

[ref123] Kade M. J.; Burke D. J.; Hawker C. J. The Power of Thiol-Ene Chemistry. J. Polym. Sci. Part A Polym. Chem. 2010, 48 (4), 743–750. 10.1002/pola.23824.

[ref124] Campos L. M.; Killops K. L.; Sakai R.; Paulusse J. M. J.; Damiron D.; Drockenmuller E.; Messmore B. W.; Hawker C. J. Development of Thermal and Photochemical Strategies for Thiol– Ene Click Polymer Functionalization. Macromolecules 2008, 41 (19), 7063–7070. 10.1021/ma801630n.

[ref125] van Geel R.; Pruijn G. J. M.; van Delft F. L.; Boelens W. C. Preventing Thiol-Yne Addition Improves the Specificity of Strain-Promoted Azide–Alkyne Cycloaddition. Bioconjugate Chem. 2012, 23 (3), 392–398. 10.1021/bc200365k. 22372991

[ref126] Wilson A.; Gasparini G.; Matile S. Functional Systems with Orthogonal Dynamic Covalent Bonds. Chem. Soc. Rev. 2014, 43 (6), 1948–1962. 10.1039/C3CS60342C. 24287608

[ref127] Orrillo A. G.; Furlan R. L. E. Sulfur in Dynamic Covalent Chemistry. Angew. Chem. 2022, 134 (26), e202201168 10.1002/ange.202201168. 35447003

[ref128] Klepel F.; Ravoo B. J. Dynamic Covalent Chemistry in Aqueous Solution by Photoinduced Radical Disulfide Metathesis. Org. Biomol. Chem. 2017, 15 (18), 3840–3842. 10.1039/C7OB00667E. 28406256

[ref129] Dawson P. E.; Muir T. W.; Clark-Lewis I.; Kent S. B. Synthesis of Proteins by Native Chemical Ligation. Science. 1994, 266 (5186), 776–779. 10.1126/science.7973629. 7973629

[ref130] Agouridas V.; El Mahdi O.; Diemer V.; Cargoët M.; Monbaliu J.-C. M.; Melnyk O. Native Chemical Ligation and Extended Methods: Mechanisms, Catalysis, Scope, and Limitations. Chem. Rev. 2019, 119 (12), 7328–7443. 10.1021/acs.chemrev.8b00712. 31050890

[ref131] Conibear A. C.; Watson E. E.; Payne R. J.; Becker C. F. W. Native Chemical Ligation in Protein Synthesis and Semi-Synthesis. Chem. Soc. Rev. 2018, 47 (24), 9046–9068. 10.1039/C8CS00573G. 30418441

[ref132] Karkas M. D. Photochemical Generation of Nitrogen-Centered Amidyl, Hydrazonyl, and Imidyl Radicals: Methodology Developments and Catalytic Applications. ACS Catal. 2017, 7 (8), 4999–5022. 10.1021/acscatal.7b01385.

[ref133] De Jager T. L.; Cockrell A. E.; Du Plessis S. S. Ultraviolet Light Induced Generation of Reactive Oxygen Species. Ultrav. Light Hum. Heal. Dis. Environ. 2017, 996, 15–23. 10.1007/978-3-319-56017-5_2. 29124687

[ref134] Brem R.; Karran P. Multiple Forms of DNA Damage Caused by UVA Photoactivation of DNA 6-thioguanine. Photochem. Photobiol. 2012, 88 (1), 5–13. 10.1111/j.1751-1097.2011.01043.x. 22077233

[ref135] Cadet J.; Mouret S.; Ravanat J.; Douki T. Photoinduced Damage to Cellular DNA: Direct and Photosensitized Reactions. Photochem. Photobiol. 2012, 88 (5), 1048–1065. 10.1111/j.1751-1097.2012.01200.x. 22780837

[ref136] Girard P. M.; Francesconi S.; Pozzebon M.; Graindorge D.; Rochette P.; Drouin R.; Sage E. UVA-Induced Damage to DNA and Proteins: Direct versus Indirect Photochemical Processes. Journal of Physics: Conference Series 2011, 261, 012002 10.1088/1742-6596/261/1/012002.

[ref137] Greenberg M. M. Pyrimidine Nucleobase Radical Reactivity in DNA and RNA. Radiat. Phys. Chem. 2016, 128, 82–91. 10.1016/j.radphyschem.2016.06.003. PMC5087805 27812242

[ref138] Song W.; Wang Y.; Qu J.; Madden M. M.; Lin Q. A Photoinducible 1, 3-dipolar Cycloaddition Reaction for Rapid, Selective Modification of Tetrazole-containing Proteins. Angew. Chem., Int. Ed. 2008, 47 (15), 2832–2835. 10.1002/anie.200705805. 18311742

[ref139] Kumar G. S.; Racioppi S.; Zurek E.; Lin Q. Superfast Tetrazole–BCN Cycloaddition Reaction for Bioorthogonal Protein Labeling on Live Cells. J. Am. Chem. Soc. 2022, 144 (1), 57–62. 10.1021/jacs.1c10354. 34964645 PMC8982153

[ref140] Zhao S.; Dai J.; Hu M.; Liu C.; Meng R.; Liu X.; Wang C.; Luo T. Photo-Induced Coupling Reactions of Tetrazoles with Carboxylic Acids in Aqueous Solution: Application in Protein Labelling. Chem. Commun. 2016, 52 (25), 4702–4705. 10.1039/C5CC10445A. 26953773

[ref141] Holland J. P.; Gut M.; Klingler S.; Fay R.; Guillou A. Photochemical Reactions in the Synthesis of Protein–Drug Conjugates. Chem.—Eur. J. 2020, 26 (1), 33–48. 10.1002/chem.201904059. 31599057

[ref142] Lee J.; Koo N.; Min D. B. Reactive Oxygen Species, Aging, and Antioxidative Nutraceuticals. Compr. Rev. Food Sci. Food Saf. 2004, 3 (1), 21–33. 10.1111/j.1541-4337.2004.tb00058.x. 33430557

[ref143] Lushchak V. I. Free Radicals, Reactive Oxygen Species, Oxidative Stress and Its Classification. Chem. Biol. Interact. 2014, 224, 164–175. 10.1016/j.cbi.2014.10.016. 25452175

[ref144] Mates J. M. Effects of Antioxidant Enzymes in the Molecular Control of Reactive Oxygen Species Toxicology. Toxicology 2000, 153 (1–3), 83–104. 10.1016/S0300-483X(00)00306-1. 11090949

[ref145] Kumar G. S.; Lin Q. Light-Triggered Click Chemistry. Chem. Rev. 2021, 121 (12), 6991–7031. 10.1021/acs.chemrev.0c00799. 33104332 PMC8071840

[ref146] Mueller J. O.; Schmidt F. G.; Blinco J. P.; Barner-Kowollik C. Visible-Light-Induced Click Chemistry. Angew. Chem., Int. Ed. 2015, 54 (35), 10284–10288. 10.1002/anie.201504716. 26179164

[ref147] Fairbanks B. D.; Macdougall L. J.; Mavila S.; Sinha J.; Kirkpatrick B. E.; Anseth K. S.; Bowman C. N. Photoclick Chemistry: A Bright Idea. Chem. Rev. 2021, 121 (12), 6915–6990. 10.1021/acs.chemrev.0c01212. 33835796 PMC9883840

[ref148] Poloukhtine A. A.; Mbua N. E.; Wolfert M. A.; Boons G.-J.; Popik V. V. Selective Labeling of Living Cells by a Photo-Triggered Click Reaction. J. Am. Chem. Soc. 2009, 131 (43), 15769–15776. 10.1021/ja9054096. 19860481 PMC2776736

[ref149] Safavi-Mirmahalleh S.-A.; Golshan M.; Gheitarani B.; Hosseini M. S.; Salami-Kalajahi M. A Review on Applications of Coumarin and Its Derivatives in Preparation of Photo-Responsive Polymers. Eur. Polym. J. 2023, 198, 112430 10.1016/j.eurpolymj.2023.112430.

[ref150] Li Z.; Qian L.; Li L.; Bernhammer J. C.; Huynh H. V.; Lee J.; Yao S. Q. Tetrazole Photoclick Chemistry: Reinvestigating Its Suitability as a Bioorthogonal Reaction and Potential Applications. Angew. Chem., Int. Ed. 2016, 55 (6), 2002–2006. 10.1002/anie.201508104. 26640085

[ref151] Benson F. R. The Chemistry of the Tetrazoles. Chem. Rev. 1947, 41 (1), 1–61. 10.1021/cr60128a001. 20257066

[ref152] Shang X.; Lai R.; Song X.; Li H.; Niu W.; Guo J. Improved Photoinduced Fluorogenic Alkene–Tetrazole Reaction for Protein Labeling. Bioconjugate Chem. 2017, 28 (11), 2859–2864. 10.1021/acs.bioconjchem.7b00562. PMC5688003 29022697

[ref153] Kuan S. L.; Wang T.; Weil T. Site-Selective Disulfide Modification of Proteins: Expanding Diversity beyond the Proteome. Chem.—Eur. J. 2016, 22 (48), 17112–17129. 10.1002/chem.201602298. 27778400 PMC5600100

[ref154] Ravasco J. M. J. M.; Faustino H.; Trindade A.; Gois P. M. P. Bioconjugation with Maleimides: A Useful Tool for Chemical Biology. Chem. - A Eur. J. 2019, 25 (1), 43–59. 10.1002/chem.201803174. 30095185

[ref155] Yu Z.; Pan Y.; Wang Z.; Wang J.; Lin Q. Genetically Encoded Cyclopropene Directs Rapid, Photoclick-chemistry-mediated Protein Labeling in Mammalian Cells. Angew. Chem. 2012, 124 (42), 10752–10756. 10.1002/ange.201205352. PMC3517012 22997015

[ref156] Feng W.; Li L.; Yang C.; Welle A.; Trapp O.; Levkin P. A. UV-Induced Tetrazole-Thiol Reaction for Polymer Conjugation and Surface Functionalization. Angew. Chem. 2015, 127 (30), 8856–8859. 10.1002/ange.201502954. 26059870

[ref157] Klán P.; Solomek T.; Bochet C. G.; Blanc A.; Givens R.; Rubina M.; Popik V.; Kostikov A.; Wirz J. Photoremovable Protecting Groups in Chemistry and Biology: Reaction Mechanisms and Efficacy. Chem. Rev. 2013, 113 (1), 119–191. 10.1021/cr300177k. 23256727 PMC3557858

[ref158] Young D. D.; Deiters A. Photochemical Control of Biological Processes. Org. Biomol. Chem. 2007, 5 (7), 999–1005. 10.1039/B616410M. 17377650

[ref159] Bochet C. G. Photolabile Protecting Groups and Linkers. J. Chem. Soc., Perkin Trans. 2002, 1 (2), 125–142. 10.1039/b009522m.

[ref160] Hansen M. J.; Velema W. A.; Lerch M. M.; Szymanski W.; Feringa B. L. Wavelength-Selective Cleavage of Photoprotecting Groups: Strategies and Applications in Dynamic Systems. Chem. Soc. Rev. 2015, 44 (11), 3358–3377. 10.1039/C5CS00118H. 25917924

[ref161] Liu J.; Kang W.; Wang W. Photocleavage-based Photoresponsive Drug Delivery. Photochem. Photobiol. 2022, 98 (2), 288–302. 10.1111/php.13570. 34861053

[ref162] Bao C.; Zhu L.; Lin Q.; Tian H. Building Biomedical Materials Using Photochemical Bond Cleavage. Adv. Mater. 2015, 27 (10), 1647–1662. 10.1002/adma.201403783. 25655424

[ref163] So W. H.; Wong C. T. T.; Xia J. Peptide Photocaging: A Brief Account of the Chemistry and Biological Applications. Chin. Chem. Lett. 2018, 29 (7), 1058–1062. 10.1016/j.cclet.2018.05.015.

[ref164] Hoffmann N. Photochemical Reactions as Key Steps in Organic Synthesis. Chem. Rev. 2008, 108 (3), 1052–1103. 10.1021/cr0680336. 18302419

[ref165] Wöll D.; Walbert S.; Stengele K.; Albert T. J.; Richmond T.; Norton J.; Singer M.; Green R. D.; Pfleiderer W.; Steiner U. E. Triplet-sensitized Photodeprotection of Oligonucleotides in Solution and on Microarray Chips. Helv. Chim. Acta 2004, 87 (1), 28–45. 10.1002/hlca.200490015.

[ref166] Hasan A.; Stengele K.-P.; Giegrich H.; Cornwell P.; Isham K. R.; Sachleben R. A.; Pfleiderer W.; Foote R. S. Photolabile Protecting Groups for Nucleosides: Synthesis and Photodeprotection Rates. Tetrahedron 1997, 53 (12), 4247–4264. 10.1016/S0040-4020(97)00154-3.

[ref167] Paul W. E. History of Interleukin-4. Cytokine 2015, 75 (1), 3–7. 10.1016/j.cyto.2015.01.038. 25814340 PMC4532601

[ref168] Gärtner Y.; Bitar L.; Zipp F.; Vogelaar C. F. Interleukin-4 as a Therapeutic Target. Pharmacol. Ther. 2023, 242, 108348 10.1016/j.pharmthera.2023.108348. 36657567

[ref169] O’Hagan M. P.; Duan Z.; Huang F.; Laps S.; Dong J.; Xia F.; Willner I. Photocleavable Ortho-Nitrobenzyl-Protected DNA Architectures and Their Applications. Chem. Rev. 2023, 123 (10), 6839–6887. 10.1021/acs.chemrev.3c00016. 37078690 PMC10214457

[ref170] Ninomiya M.; Egholm C.; Breu D.; Boyman O.; Bode J. In Vitro and in Vivo Evaluation of Chemically Synthesized, Receptor-Biased Interleukin-4 and Photocaged Variants. ChemRxiv Preprint 2024, 10.26434/chemrxiv-2024-s31dq. PMC12190007 40561016

[ref171] Roth P.; Meyer R.; Harley I.; Landfester K.; Lieberwirth I.; Wagner M.; Ng D. Y. W.; Weil T. Supramolecular Assembly Guided by Photolytic Redox Cycling. Nat. Synth. 2023, 2 (10), 980–988. 10.1038/s44160-023-00343-1.

[ref172] Ellis M. K.; Hill S.; Foster P. M. D. Reactions of Nitrosonitrobenzenes with Biological Thiols: Identification and Reactivity of Glutathion-S-Yl Conjugates. Chem. Biol. Interact. 1992, 82 (2), 151–163. 10.1016/0009-2797(92)90107-V. 1568267

[ref173] Wainwright M. The Use of Dyes in Modern Biomedicine. Biotechnol. Histochem. 2003, 78 (3–4), 147–155. 10.1080/10520290310001602404. 14714878

[ref174] Demchenko A. P. Photobleaching of Organic Fluorophores: Quantitative Characterization, Mechanisms, Protection. Methods Appl. Fluoresc. 2020, 8 (2), 022001 10.1088/2050-6120/ab7365. 32028269

[ref175] Valeur B.; Brochon J.-C. New Trends in Fluorescence Spectroscopy: Applications to Chemical and Life Sciences; Springer Science & Business Media, 2012; Vol. 1.

[ref176] Kwon J.; Elgawish M. S.; Shim S. Bleaching-Resistant Super-Resolution Fluorescence Microscopy. Adv. Sci. 2022, 9 (9), 2101817 10.1002/advs.202101817. PMC8948665 35088584

[ref177] Lac A.; Le Lam A.; Heit B. Optimizing Long-Term Live Cell Imaging. Fluorescent Microscopy; Springer, 2022; pp 57–73. 10.1007/978-1-0716-2051-9_3 35218532

[ref178] Spikes J. D.; MacKnight M. L. Dye-sensitized Photooxidation of Proteins. Ann. N.Y. Acad. Sci. 1970, 171 (1), 149–162. 10.1111/j.1749-6632.1970.tb39319.x.

[ref179] Byers G. W.; Gross S.; Henrichs P. M. Direct and Sensitized Photooxidation of Cyanine Dyes. Photochem. Photobiol. 1976, 23 (1), 37–43. 10.1111/j.1751-1097.1976.tb06768.x. 1265127

[ref180] Gopika G. S.; Prasad P. M. H.; Lekshmi A. G.; Lekshmypriya S.; Sreesaila S.; Arunima C.; Kumar M. S.; Anil A.; Sreekumar A.; Pillai Z. S. Chemistry of Cyanine Dyes-A Review. Mater. Today Proc. 2021, 46, 3102–3108. 10.1016/j.matpr.2021.02.622.

[ref181] Shindy H. A. Fundamentals in the Chemistry of Cyanine Dyes: A Review. Dye. Pigment. 2017, 145, 505–513. 10.1016/j.dyepig.2017.06.029.

[ref182] Pasdaran A.; Zare M.; Hamedi A.; Hamedi A. A Review of the Chemistry and Biological Activities of Natural Colorants, Dyes, and Pigments: Challenges, and Opportunities for Food, Cosmetics, and Pharmaceutical Application. Chem. Biodivers. 2023, 20 (8), e202300561 10.1002/cbdv.202300561. 37471105

[ref183] Köckenberger J.; Klemt I.; Sauer C.; Arkhypov A.; Reshetnikov V.; Mokhir A.; Heinrich M. R. Cyanine-and Rhodamine-Derived Alkynes for the Selective Targeting of Cancerous Mitochondria through Radical Thiol-Yne Coupling in Live Cells. Chem.—Eur. J. 2023, 29 (45), e202301340 10.1002/chem.202301340. 37171462

[ref184] Gidi Y.; Payne L.; Glembockyte V.; Michie M. S.; Schnermann M. J.; Cosa G. Unifying Mechanism for Thiol-Induced Photoswitching and Photostability of Cyanine Dyes. J. Am. Chem. Soc. 2020, 142 (29), 12681–12689. 10.1021/jacs.0c03786. 32594743 PMC8500274

[ref185] Dempsey G. T.; Bates M.; Kowtoniuk W. E.; Liu D. R.; Tsien R. Y.; Zhuang X. Photoswitching Mechanism of Cyanine Dyes. J. Am. Chem. Soc. 2009, 131 (51), 18192–18193. 10.1021/ja904588g. 19961226 PMC2797371

[ref186] Vaughan J. C.; Dempsey G. T.; Sun E.; Zhuang X. Phosphine Quenching of Cyanine Dyes as a Versatile Tool for Fluorescence Microscopy. J. Am. Chem. Soc. 2013, 135 (4), 1197–1200. 10.1021/ja3105279. 23311875 PMC3624894

